# National trends and disparities in coronary artery disease and depression-related mortality in the United States, 1999–2025: A population-based CDC WONDER analysis

**DOI:** 10.1016/j.ahjo.2026.100936

**Published:** 2026-10-07

**Authors:** Hassaan Abid, Sohana Memon, Vishan Das, Tauseef Hamid, Kaneez Fatima, Muhammad Mukhlis, Muhammad Vazaym, Rithik Kumar Khiani

**Affiliations:** a Department of Medicine, Indiana University School of Medicine, Muncie, Indiana, USA; b Department of Medicine, Liaquat University of Medical and Health Sciences (LUMHS), Jamshoro, Pakistan; c Department of Medicine, Saidu Medical College, Swat, Pakistan; d Department of Medicine, CMH Institute of Medical Science, Multan, Pakistan; e Department of Medicine, Ayub Medical College, Abbottabad, Pakistan; f Department of Medicine, Jinnah Sindh Medical University, Rafiqi H J Shaheed Road, Karachi, Pakistan

**Keywords:** Coronary artery disease, Depressive disorders, Mortality trends, Age-adjusted mortality rate, CDC WONDER

## Abstract

**Background:**

Coronary artery disease (CAD) and depressive disorders frequently coexist, but national mortality patterns involving both conditions remain incompletely characterized.

**Objective:**

To evaluate temporal trends and demographic and geographic disparities in mortality involving CAD and depressive or depressive-anxiety disorders in the United States from 1999 to 2025.

**Methods:**

We analyzed U.S. residents aged ≥25 years using the CDC WONDER Multiple Cause of Death database. Deaths were included when CAD (ICD-10 I20–I25) and a depressive or depressive-anxiety disorder (F32, F33, or F41.2) were both recorded on the same death certificate, irrespective of underlying or contributing cause. Age-adjusted mortality rates (AAMRs) were standardized to the 2000 U.S. population, and temporal trends were assessed using Joinpoint regression. Data were extracted April 10, 2026; 2025 mortality data were provisional. Urbanization analyses were limited to 1999–2020.

**Results:**

Among 85,806 deaths, the mean annual AAMR was 1.42 per 100,000 population. AAMR declined from 1.81 (95% CI, 1.75–1.87) in 1999 to 1.31 (95% CI, 1.26–1.35) in 2025 (AAPC, −1.38%; 95% CI, −2.24 to −0.50; *p* = 0.002), despite an increase during 2017–2020. Mean annual AAMRs were higher among males than females (1.52 vs 1.33), White individuals (1.59), the Midwest (1.91), and nonmetropolitan versus metropolitan areas during 1999–2020 (1.94 vs 1.33). Mortality increased among adults aged 35–44 and 55–64 years and among Black individuals from 2014 to 2025.

**Conclusions:**

Mortality involving both conditions declined overall but varied substantially across demographic and geographic subgroups, with increases in some younger and middle-aged populations.

## Graphical abstract

**Figure:**
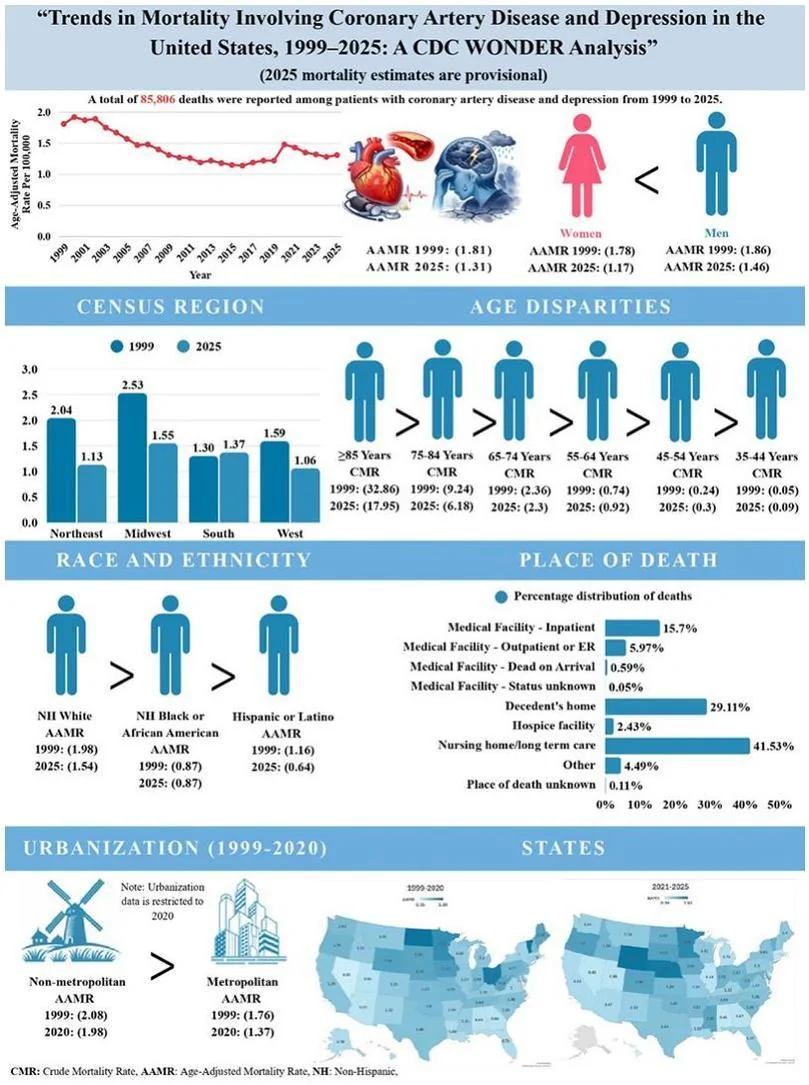

## 1 Introduction

Coronary artery disease (CAD) remains the leading cause of mortality globally and a major contributor to cardiovascular burden in the United States, accounting for a substantial proportion of deaths despite advances in prevention and treatment strategies [1], [2], [3]. According to the Global Burden of Disease Study, cardiovascular diseases collectively accounted for 18.6 million deaths globally, with ischemic heart disease representing the single largest contributor [3]. Although age-adjusted mortality rates from CAD declined over previous decades, recent evidence suggests a plateauing of this trend, accompanied by persistent disparities across demographic and geographic subgroups [4].

Depression is a common and increasingly prevalent mental health condition among adults in the United States, with recent national data indicating a rising burden of depressive symptoms [5]. A substantial body of literature has demonstrated that depression is associated with adverse cardiovascular outcomes, including increased risk of incident CAD, poorer prognosis among individuals with established disease, and higher mortality [1], [6], [7], [8], [9]. This relationship is likely multifactorial, involving behavioral pathways such as reduced adherence to medical therapy and unhealthy lifestyle patterns, as well as biological mechanisms including systemic inflammation and neuroendocrine dysregulation.

Depression has been associated with an increased risk of CAD through these behavioral and biological pathways, while depressive symptoms may also develop or worsen in the context of established CAD because of physical limitations and psychosocial burden. Consequently, the two conditions may coexist frequently within affected populations [10]. However, these clinical associations should not be interpreted as evidence of a temporal or causal relationship in mortality data, because death-certificate records cannot establish disease sequence, direct contribution to death, or underlying mechanistic pathways between CAD and depression.

Despite the recognized association between CAD and depression, population-level evidence describing mortality involving both conditions remains limited. Prior national and CDC WONDER-based studies have examined CAD-related or depression-related mortality separately, while mortality trends specifically involving the co-occurrence of CAD and depression on the same death certificate remain less well characterized. In particular, comprehensive evaluations of long-term temporal trends and disparities across sex, race and ethnicity, age, and geographic regions are limited. The present study addresses this gap by focusing specifically on deaths in which both CAD and a qualifying depressive or depressive-anxiety disorder were recorded, incorporating detailed demographic and geographic subgroup analyses, and extending the evaluation through 2025 to characterize recent national mortality patterns, with 2025 observations based on provisional mortality data.

This study examined temporal trends and demographic and geographic disparities in mortality involving CAD and depression among U.S. adults aged ≥25 years using the CDC WONDER Multiple Cause of Death database. By evaluating age-adjusted mortality rates over more than two decades, we sought to characterize the evolving national mortality burden and identify heterogeneity in mortality patterns across sex, age, race and ethnicity, and geographic subgroups. The findings are intended to inform mortality surveillance and generate hypotheses for further patient-level research into the clinical and healthcare factors underlying these observed patterns.

## 2 Methods

### 2.1 Study design and data source

This descriptive, retrospective, population-based study evaluated mortality trends among U.S. residents aged ≥25 years from 1999 through 2025. Mortality data were obtained from the Centers for Disease Control and Prevention Wide-Ranging Online Data for Epidemiologic Research (CDC WONDER) Multiple Cause of Death (MCD) database [11]. Data were extracted on 10 April 2026. The CDC WONDER MCD database provides death-certificate information for U.S. residents, including the underlying cause of death and multiple contributing causes recorded on each death certificate. Because the present study identified deaths in which both coronary artery disease (CAD) and a qualifying depressive or depressive-anxiety disorder were recorded, the multiple-cause-of-death fields were used rather than restricting case identification to the underlying cause of death.

Final mortality data were used for calendar years for which final records were available, whereas observations for **2025 were obtained from the CDC WONDER provisional Multiple Cause of Death database** and therefore represent provisional mortality records available as of 10 April 2026. Provisional mortality records may be incomplete and are subject to subsequent revision as additional death certificates are received, processed, and coded by the National Center for Health Statistics (NCHS).

CAD was defined using International Classification of Diseases, Tenth Revision (ICD-10) codes **I20–I25**. The primary depression-related case definition included **F32 (depressive episodes), F33 (recurrent depressive disorder), and F41.2 (mixed anxiety and depressive disorder)**. The inclusion of these codes was based on precedent from previously published mortality studies using similar ICD-10 definitions [12], [13].

Because F41.2 represents a mixed anxiety-depressive disorder rather than a depressive disorder alone, the broader terminology **“depressive or depressive-anxiety disorder”** is used throughout the manuscript when referring to the case definition. A death was included only when both CAD and a qualifying depressive or depressive-anxiety disorder were recorded on the **same death certificate** in the multiple-cause-of-death fields, irrespective of which condition was designated as the underlying cause of death. Thus, the analysis identifies deaths involving both conditions and does not establish disease sequence, temporal ordering, causality, or a bidirectional relationship between CAD and depressive disorders.

A sensitivity analysis excluding F41.2 was performed to evaluate whether inclusion of mixed anxiety and depressive disorder materially affected the observed mortality patterns. The sensitivity analysis retained the same CAD definition, population restrictions, multiple-cause-of-death approach, and statistical procedures as the primary analysis, with the depressive disorder definition restricted to F32 and F33. A separate sensitivity analysis was also performed excluding the provisional 2025 mortality data, restricting the analysis to 1999–2024 final mortality records, to assess the robustness of the observed temporal trends to the inclusion of provisional observations.

CDC WONDER mortality data are de-identified and publicly available; therefore, institutional review board approval was not required. The study was conducted and reported in accordance with the Strengthening the Reporting of Observational Studies in Epidemiology (STROBE) guidelines for observational research [14].

### 2.2 Study population and variables

The study population comprised deaths among U.S. residents aged **≥25 years** from 1999 through 2025. The analysis included residents of all 50 U.S. states and the District of Columbia. Non-U.S. residents were excluded through the CDC WONDER residence-based mortality framework. For age-specific analyses, crude mortality rates (CMRs) were calculated separately within each age group and used for Joinpoint regression. Age-specific analyses included the 35–44, 45–54, 55–64, 65–74, 75–84, and ≥ 85 years groups; the 25–34-year group was excluded because the corresponding CDC WONDER estimates did not meet reliability criteria. Joinpoint models were fitted using the underlying unrounded annual mortality rates rather than rounded values. Sex was classified as male or female according to the death certificate. Race and ethnicity were categorized according to the classifications available in the respective CDC WONDER mortality files. Where applicable, analyses using bridged-race mortality files used the corresponding bridged-race classifications, whereas analyses using single-race mortality files used the respective CDC WONDER single-race classifications. The racial and ethnic categories included non-Hispanic White, non-Hispanic Black or African American, non-Hispanic American Indian or Alaska Native, non-Hispanic Asian or Pacific Islander, and Hispanic or Latino.

Estimates for non-Hispanic American Indian or Alaska Native and non-Hispanic Asian or Pacific Islander populations were suppressed by CDC WONDER and therefore were not reported as numerical estimates or included in comparative interpretation.

U.S. Census regions were categorized as Northeast, Midwest, South, and West. State-level mortality data were extracted for geographic comparisons. Place of death was categorized according to the categories available in the CDC WONDER mortality database. Urbanization was assessed using the **National Center for Health Statistics (NCHS) urban-rural classification scheme** available for the 1999–2020 mortality data used in this analysis. Accordingly, urbanization analyses were restricted to **1999–2020** and were not extended to 2021–2025.

### 2.3 Statistical analysis

Crude mortality rates and age-adjusted mortality rates (AAMRs) per **100,000 population** were calculated using the standard CDC WONDER methodology. AAMRs were directly standardized to the **2000 U.S. standard population** to facilitate comparisons across calendar years and demographic strata. Temporal trends in mortality were evaluated using **Joinpoint Regression Program version 5.4** [15]. Joinpoint regression was used to identify statistically significant changes in the direction and magnitude of mortality trends over time. **Annual percent change (APC)** was estimated for each identified trend segment, while **average annual percent change (AAPC)** summarized the overall temporal trend across the specified study period. Corresponding 95% confidence intervals (CIs) were reported. Statistical significance was assessed using two-sided tests, with ***p*** **<** **0.05** considered statistically significant.

The **Monte Carlo permutation method** was used for model selection and to evaluate statistically significant changes in trend. A maximum of **four joinpoints** was permitted for each temporal analysis. Joinpoint regression models were fitted using the annual mortality-rate estimates and their corresponding standard errors. A **logarithmic transformation** of the rates was used, with the models accounting for **heteroscedasticity** through the standard error-based modeling approach. An **uncorrelated error structure** was specified. The final number and locations of joinpoints were determined using the permutation-based model-selection procedure implemented in Joinpoint Regression Program version 5.4.

CDC WONDER confidentiality and data-suppression criteria were applied to the extracted mortality estimates. Estimates subject to CDC WONDER suppression were not reported as numerical values and were identified as suppressed in the relevant tables or supplementary materials.

## 3 Results

### 3.1 Overall

The overall population had a mean annual AAMR of 1.42, with a total of 85,806 deaths. By sex males had a higher mean annual AAMR (1.52) with 37,875 deaths compared with females, who had a mean annual AAMR of 1.33 and 47,931 deaths. In the overall population, AAMRs declined from 1.81 (95% CI: 1.75–1.87) in 1999 to 1.31 (95% CI: 1.26–1.35) in 2025 (AAPC: −1.38; 95% CI: −2.24 to −0.50, *p* = 0.002). Mortality rates were stable between 1999 and 2001, followed by a significant decline from 2001 to 2010 (APC: −4.58; 95% CI: −5.33 to −3.83, *p* < 0.001) and a continued decrease through 2017 (APC: −1.52; 95% CI: −2.57 to −0.46, *p* = 0.009). Mortality rates then showed a notable increase between 2017 and 2020 (APC: 8.09; 95% CI: 1.60–14.99, *p* = 0.018), before declining significantly from 2020 to 2025 (APC: −2.21; 95% CI: −3.42 to −0.99, *p* = 0.002). As illustrated in, Table 1 Supplemental **Table 1,** Fig. 1.

**Table 1:** Deaths, age-adjusted mortality rates (AAMR), and temporal trends annual percent change [APC] and average annual percent change [AAPC] of coronary artery disease and depression related deaths among U.S. adults aged ≥25 years, 1999–2025.

Year	APC (95% CI)	*p*-value for APC	AAPC (95% CI)	p-value for AAPC	Deaths	Mean Age-Adjusted Mortality Rate (95% CI)
**Overall**						
1999 to 2001	2.43 (−3.61 to 8.85)	0.409	−1.38 (−2.24 to −0.50)	0.002	85,806	1.42 (1.37–1.47)
2001 to 2010	−4.58 (−5.33 to −3.83)	<0.001				
2010 to 2017	−1.52 (−2.57 to −0.46)	0.009				
2017 to 2020	8.09 (1.60 to 14.99)	0.018				
2020 to 2025	−2.21 (−3.42 to −0.99)	0.002				
**Sex**						
**Women**						
1999 to 2001	1.59 (−5.58 to 9.30)	0.649	−1.81 (−3.01 to −0.60)	0.003	47,931	1.33 (1.27–1.4)
2001 to 2010	−4.87 (−5.66 to −4.06)	<0.001				
2010 to 2017	−2.13 (−3.64 to −0.60)	0.010				
2017 to 2020	7.74 (−1.74 to 18.14)	0.104				
2020 to 2025	−2.60 (−4.40 to −0.76)	0.009				
**Men**						
1999 to 2002	1.17 (−1.93 to 4.37)	0.435	−0.92 (−1.80 to −0.03)	0.043	37,875	1.52 (1.44–1.6)
2002 to 2010	−4.33 (−5.15 to −3.51)	<0.001				
2010 to 2017	−0.91 (−2.08 to 0.27)	0.119				
2017 to 2020	8.01 (0.99 to 15.53)	0.028				
2020 to 2025	−1.74 (−3.06 to −0.40)	0.015				
**Race** (Estimate for Non-Hispanic American Indian/Alaska Native and non-Hispanic Asian /pacific islanders were suppressed and considered statistically unreliable.						
**NH Black or African American**						
1999 to 2014	−2.41 (−4.20 to −0.59)	0.012	0.21 (−1.19 to 1.62)	0.773	4395	0.78 (0.66–0.91)
2014 to 2025	3.89 (1.39 to 6.46)	0.004				
**NH White**						
1999 to 2001	2.74 (−4.01 to 9.96)	0.406	−1.09 (−1.93 to −0.24)	0.012	76,654	1.59 (1.53–1.65)
2001 to 2009	−4.57 (−5.42 to −3.72)	<0.001				
2009 to 2016	−2.07 (−3.34 to −0.80)	0.004				
2016 to 2020	6.18 (2.25 to 10.27)	0.004				
**Hispanic or Latino**						
1999 to 2025	−0.31 (−1.01 to 0.39)	0.372	−0.31 (−1.01 to 0.39)	0.372	3473	0.76 (0.62–0.92)
**Age group**						
**35–44 Years**						
1999 to 2025	0.83 (0.14 to 1.53)	0.021	0.83 (0.14 to 1.53)	0.021	905	0.08 (0.06–0.11)
**45–54 Years**						
1999–2004	8.30 (0.73 to 16.43)	0.033	1.42 (−0.02 to 2.87)	0.054	3549	0.31 (0.26–0.37)
2004–2025	−0.16 (−0.88 to 0.57)	0.657				
**55–64 Years**						
1999–2025	1.21 (0.85 to 1.58)	< 0.001	1.21 (0.85 to 1.58)	< 0.001	8735	0.88 (0.79–0.98)
**65–74 Years**						
1999–2011	−2.89 (−4.52 to −1.23)	0.002	−0.10 (−1.02 to 0.84)	0.841	14,577	2.13 (1.95–2.32)
2011–2025	2.36 (1.23 to 3.51)	< 0.001				
**75–84 Years**						
1999–2002	0.28 (−4.77 to 5.59)	0.910	−1.67 (−3.03 to −0.29)	0.018	24,965	6.51 (6.09–6.93)
2002–2006	−7.76 (−12.65 to −2.61)	0.007				
2006–2016	−3.53 (−4.63 to −2.42)	< 0.001				
2016–2020	6.91 (0.54 to 13.67)	0.035				
2020–2025	−0.60 (−2.93 to 1.78)	0.59				
**85+ Years**						
1999–2001	1.08 (−9.70 to 13.15)	0.842	−2.39 (−3.58 to −1.18)	< 0.001	32,895	22.65 (21.39–23.91)
2001–2015	−5.30 (−5.90 to −4.70)	< 0.001				
2015–2020	4.99 (0.84 to 9.31)	0.021				
2020–2025	−2.59 (−5.26 to 0.16)	0.063				
**Census region**						
**Northeast**						
1999 to 2008	−6.47 (−8.42 to −4.47)	<0.001	−2.04 (−2.89 to −1.18)	<0.001	16,472	1.36 (1.25–1.47)
2008 to 2025	0.39 (−0.48 to 1.27)	0.362				
**Midwest**						
1999 to 2007	−2.40 (−4.07 to −0.70)	0.008	−2.05 (−3.22 to −0.86)	<0.001	25,868	1.91 (1.79–2.03)
2007 to 2012	−7.60 (−12.65 to −2.26)	0.008				
2012 to 2025	0.40 (−0.48 to 1.29)	0.351				
**South**						
1999 to 2001	6.55 (−7.00 to 22.09)	0.332	−0.05 (−1.82 to 1.74)	0.953	27,145	1.22 (1.14–1.29)
2001 to 2007	−5.68 (−8.45 to −2.83)	<0.001				
2007 to 2017	−1.15 (−2.31 to 0.03)	0.055				
2017 to 2020	14.41 (1.64 to 28.79)	0.029				
2020 to 2025	−1.56 (−3.92 to 0.86)	0.185				
**West**						
1999 to 2015	−2.68 (−3.26 to −2.10)	<0.001	−1.69 (−2.19 to −1.18)	<0.001	16,321	1.26 (1.16–1.36)
2015 to 2025	−0.09 (−1.12 to 0.96)	0.867				
**Urbanization**						
**Metropolitan**						
1999 to 2011	−4.49 (−5.22 to −3.75)	<0.001	−1.74 (−2.75 to −0.71)	<0.001	51,384	1.33 (1.28–1.39)
2011 to 2018	−0.67 (−2.38 to 1.07)	0.423				
2018 to 2020	12.16 (2.34 to 22.92)	0.018				
**Non-metropolitan**						
1999 to 2016	−2.26 (−2.83 to −1.67)	<0.001	−1.16 (−2.15 to −0.17)	0.022	16,165	1.94 (1.8–2.08)
2016 to 2020	3.62 (−1.49 to 9.00)	0.156				

(Note: Urbanization data is restricted to 1999–2020.)

**Fig. 1:**
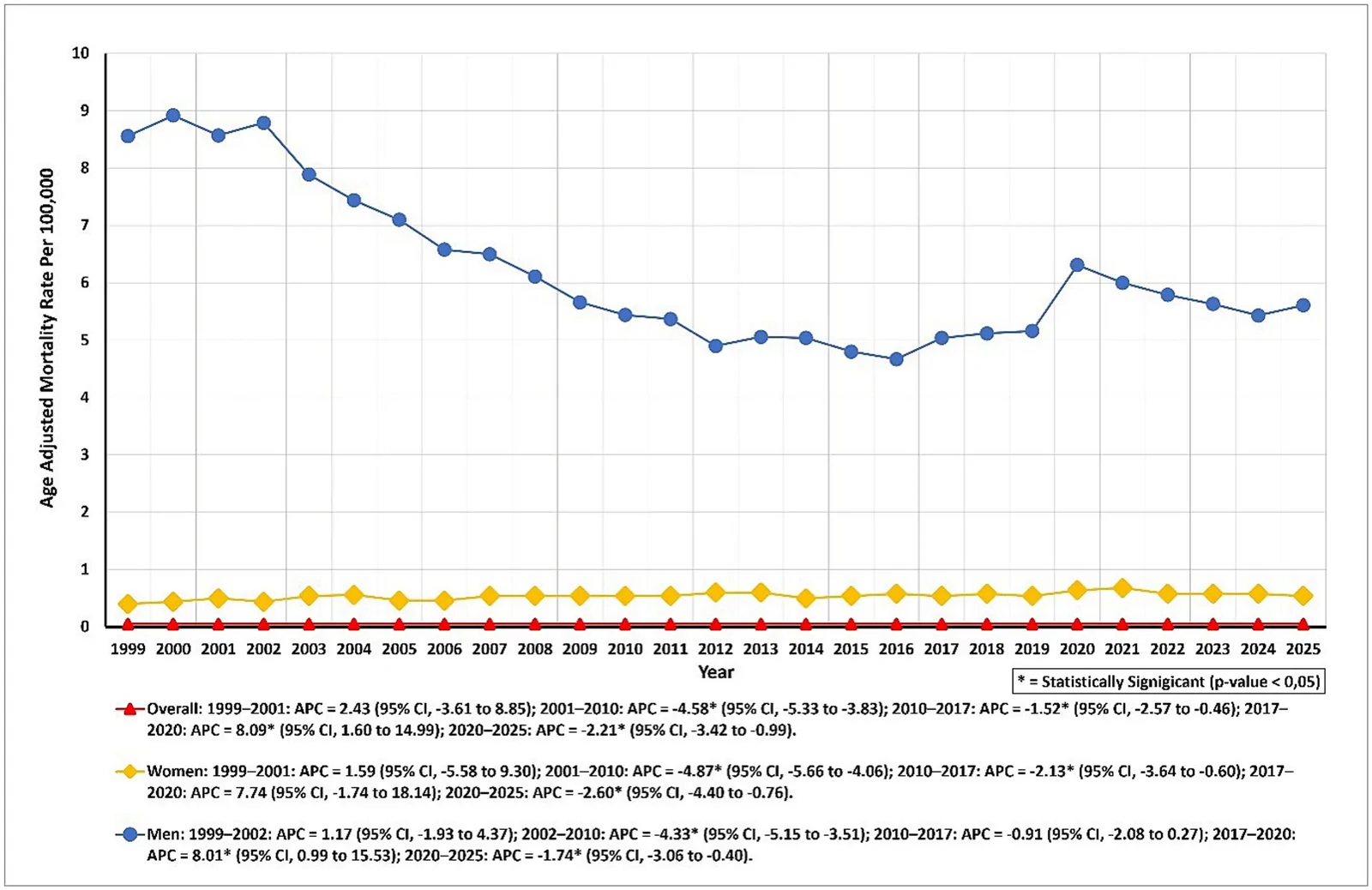
Overall and sex-stratified age-adjusted mortality rates (AAMRs) for deaths involving coronary artery disease and depressive or depressive-anxiety disorders among U.S. adults aged ≥25 years, 1999–2025. APC = Annual Percentage Change, CI = Confidence Interval. *Indicates that the Annual Percentage Change (APC) is significantly different from zero at α = 0.05.

### 3.2 Gender

In females, AAMRs declined from 1.78 (95% CI: 1.71–1.86) in 1999 to 1.17 (95% CI: 1.12–1.23) in 2025 (AAPC: −1.81; 95% CI: −3.01 to −0.60, *p* = 0.003). Mortality rates remained stable from 1999 to 2001, followed by a significant decline between 2001 and 2010 (APC: −4.87; 95% CI: −5.66 to −4.06, *p* < 0.001) and a continued decrease through 2017 (APC: −2.13; 95% CI: −3.64 to −0.60, *p* = 0.010). Mortality rates increased non-significantly between 2017 and 2020, and notable decline in rates was observed from 2020 to 2025 (APC: −2.60; 95% CI: −4.40 to −0.76, *p* = 0.009). In males, AAMRs declined from 1.86 (95% CI: 1.75–1.97) in 1999 to 1.46 (95% CI: 1.40–1.53) in 2025 (AAPC: −0.92; 95% CI: −1.80 to −0.03, *p* = 0.043). Mortality rates were stable between 1999 and 2002, followed by a significant decline from 2002 to 2010 (APC: −4.33; 95% CI: −5.15 to −3.51, *p* < 0.001). Rates remained relatively stable between 2010 and 2017, before increasing notably from 2017 to 2020 (APC: 8.01; 95% CI: 0.99–15.53, *p* = 0.028). Mortality rates subsequently declined significantly between 2020 and 2025 (APC: −1.74; 95% CI: −3.06 to −0.40, *p* = 0.015). As illustrated in, Table 1, **Supplemental Table 1,** Fig. 1.

### 3.3 Age

The mean annual CMRs were highest among the individuals aged ≥85 years age group (22.65), followed by 75–84 years (6.51), 65–74 years (2.13), 55–64 years (0.88), 45–54 years (0.31), and 35–44 years (0.08).

For the 35–44 years age group, CMRs increased significantly from 0.05 (95% CI: 0.03–0.07) in 1999 to 0.09 (95% CI: 0.06–0.12) in 2025 (AAPC: 0.83; 95% CI: 0.14–1.53, *p* = 0.021).

For the 45–54 years age group, CMRs increased from 0.24 (95% CI: 0.20–0.30) in 1999 to 0.30 (95% CI: 0.25–0.36) in 2025 (AAPC: 1.42; 95% CI: −0.02–2.87, *p* = 0.054), with a significant increase from 1999 to 2004 (APC: 8.30; 95% CI: 0.73–16.43, *p* = 0.033), followed by a non-significant decline from 2004 to 2025 (APC: −0.16; 95% CI: −0.88–0.57, *p* = 0.657).

For the 55–64 years age group, mortality rates increased markedly from 0.74 (95% CI: 0.63–0.85) in 1999 to 0.92 (95% CI: 0.83–1.02) in 2025 (AAPC: 1.21; 95% CI: 0.85–1.58, *p* < 0.001).

For the 65–74 years age group, mortality rates decreased from 2.36 (95% CI: 2.14–2.58) in 1999 to 2.30 (95% CI: 2.14–2.46) in 2025 (AAPC: −0.10; 95% CI: −1.02–0.84, *p* = 0.841), declining significantly from 1999 to 2011 (APC: −2.89; 95% CI: −4.52 to −1.23, *p* = 0.002) before increasing significantly from 2011 to 2025 (APC: 2.36; 95% CI: 1.23–3.51, *p* < 0.001).

For the 75–84 years age group, mortality rates decreased from 9.24 (95% CI: 8.70–9.77) in 1999 to 6.18 (95% CI: 5.82–6.53) in 2025 (AAPC: −1.67; 95% CI: −3.03 to −0.29, *p* = 0.018), with significant declines from 2002 to 2016 (APC: −3.53; 95% CI: −4.63 to −2.42, *p* < 0.001), followed by a significant increase from 2016 to 2020 (APC: 6.91; 95% CI: 0.54–13.67, *p* = 0.035) and a nonsignificant decline from 2020 to 2025 (APC: −0.60; 95% CI: −2.93–1.78, *p* = 0.590).

For the ≥85 years age group, mortality rates decreased from 32.86 (95% CI: 31.12–34.60) in 1999 to 17.95 (95% CI: 16.91–18.99) in 2025 (AAPC: −2.39; 95% CI: −3.58 to −1.18, *p* < 0.001), declining significantly from 2001 to 2015 (APC: −5.30; 95% CI: −5.90 to −4.70, *p* < 0.001), increasing notably from 2015 to 2020 (APC: 4.99; 95% CI: 0.84–9.31, *p* = 0.021), and then declining non significantly from 2020 to 2025 (APC: −2.59; 95% CI: −5.26–0.16, *p* = 0.063). Table 1**, Supplementary Table 2,** Fig. 2.

**Fig. 2:**
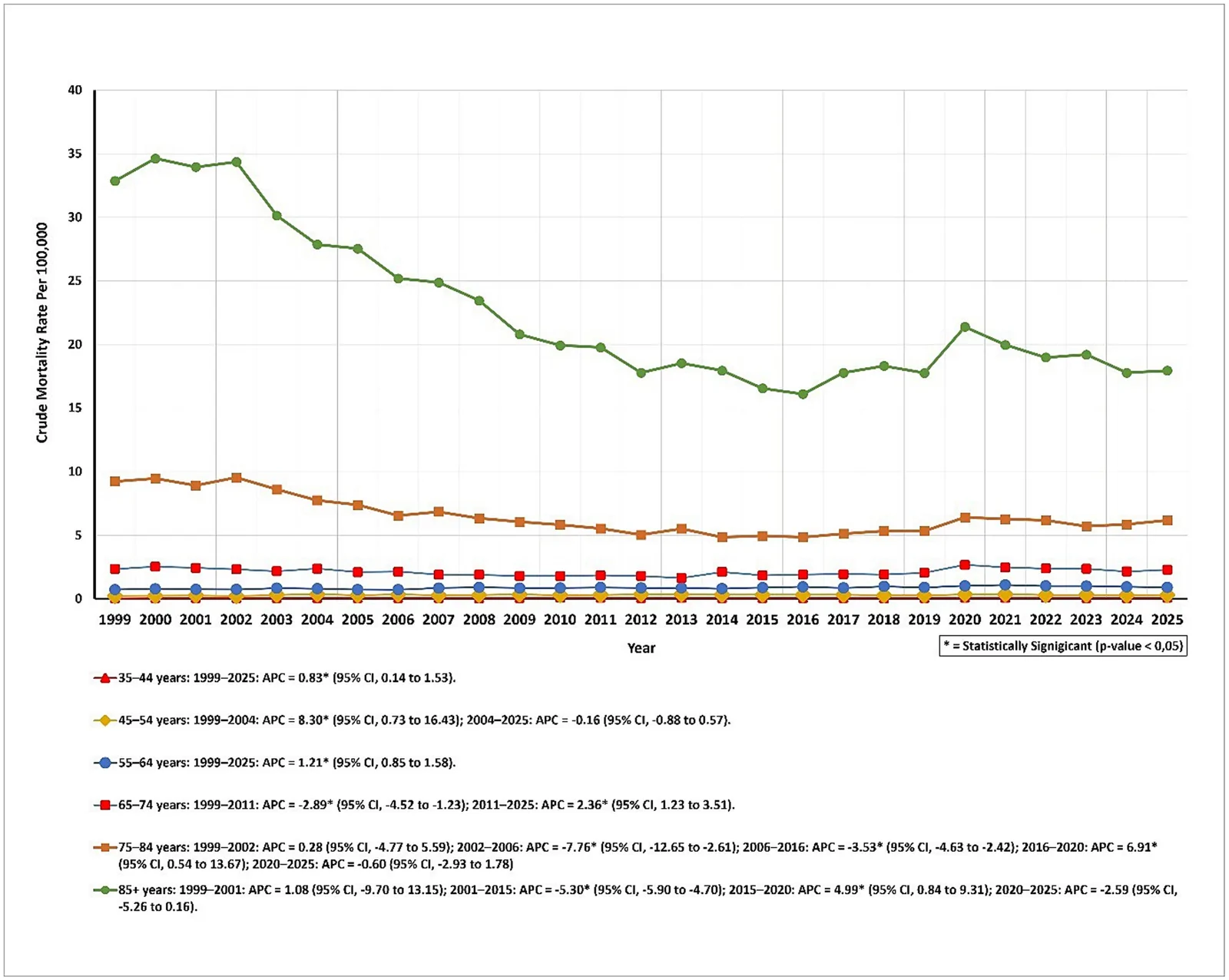
Age-specific crude mortality rates (CMRs) per 100,000 for deaths involving coronary artery disease and depressive or depressive-anxiety disorders among U.S. adults aged 35–44, 45–54, 55–64, 65–74, 75–84, and ≥ 85 years, 1999–2025. APC = Annual Percentage Change, CI = Confidence Interval. *Indicates that the Annual Percentage Change (APC) is significantly different from zero at α = 0.05.

### 3.4 Census region

Mean annual AAMRs were highest in the Midwest region (1.91), followed by the Northeast (1.36), West (1.26), and South (1.22) regions. In the Northeast region, AAMRs declined from 2.04 (95% CI: 1.90–2.19) in 1999 to 1.13 (95% CI: 1.04–1.23) in 2025 (AAPC: −2.04; 95% CI: −2.89 to −1.18, *p* < 0.001). Mortality rates decreased significantly from 1999 to 2008 (APC: −6.47; 95% CI: −8.42 to −4.47, *p* < 0.001), followed by a stable trend from 2008 to 2025. In the Midwest region, AAMRs declined from 2.53 (95% CI: 2.38–2.68) in 1999 to 1.55 (95% CI: 1.45–1.66) in 2025 (AAPC: −2.05; 95% CI: −3.22 to −0.86, *p* = 0.001). Mortality rates showed a significant decline from 1999 to 2007 (APC: −2.40; 95% CI: −4.07 to −0.70, *p* = 0.008), followed by a steeper decrease between 2007 and 2012 (APC: −7.60; 95% CI: −12.65 to −2.26, *p* = 0.008). From 2012 to 2025, rates remained relatively stable. In the South region, AAMRs increased slightly from 1.30 (95% CI: 1.21–1.39) in 1999 to 1.37 (95% CI: 1.30–1.45) in 2025 (AAPC: −0.05; 95% CI: −1.82 to 1.74, *p* = 0.953). Mortality rates were stable from 1999 to 2001, followed by a significant decline between 2001 and 2007 (APC: −5.68; 95% CI: −8.45 to −2.83, *p* = 0.001). Rates continued to decline non-significantly from 2007 to 2017, before increasing significantly from 2017 to 2020 (APC: 14.41; 95% CI: 1.64–28.79, *p* = 0.029). From 2020 to 2025, mortality rates were stable. In the West region, AAMRs declined from 1.59 (95% CI: 1.46–1.72) in 1999 to 1.06 (95% CI: 0.98–1.15) in 2025 (AAPC: −1.69; 95% CI: −2.19 to −1.18, *p* < 0.001). Mortality rates declined significantly from 1999 to 2015 (APC: −2.68; 95% CI: −3.26 to −2.10, *p* < 0.001), followed by stable trends from 2015 to 2025. As illustrated in Table 1, **Supplemental Table 3,** Fig. 3.

**Fig. 3:**
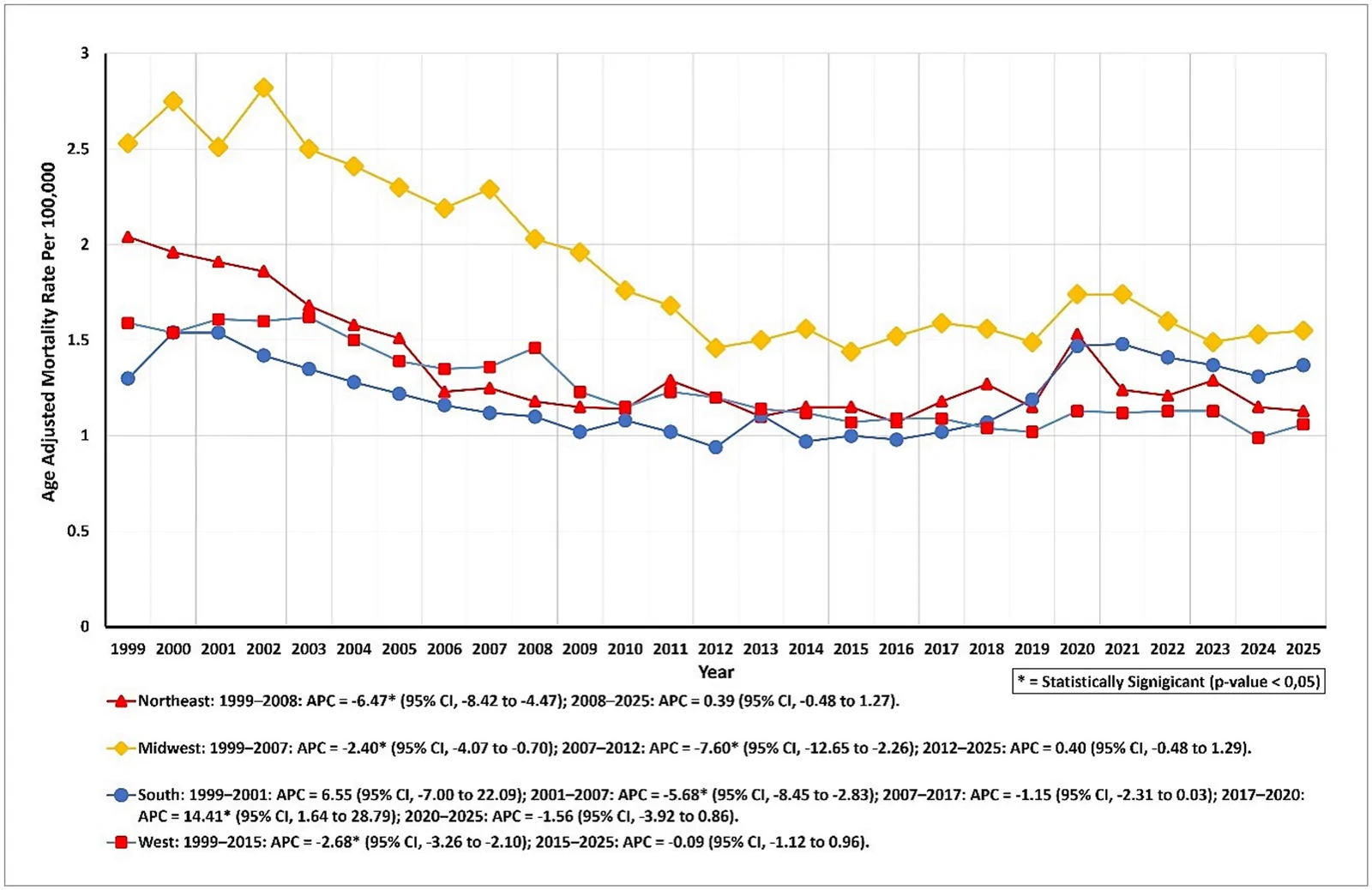
Census region stratified age-adjusted mortality rates per 100,000 for coronary artery disease and depression or depressive-anxiety disorders related mortality in United States adults aged ≥25 years, 1999–2025. APC = Annual Percentage Change, CI = Confidence Interval. *Indicates that the Annual Percentage Change (APC) is significantly different from zero at α = 0.05.

### 3.5 Race

The White population had the highest mean annual AAMR (1.59), followed by the Black or African American population (0.78) and the Hispanic and Latino population (0.76). In the Hispanic or Latino population, AAMRs declined non-significantly from 1.16 (95% CI: 0.92–1.44) in 1999 to 0.64 (95% CI: 0.55–0.75) in 2025. In the Black or African American population, AAMRs were 0.87 (95% CI: 0.72–1.03) in 1999 and 0.87 (95% CI: 0.76–0.99) in 2025, with no significant overall change during the study period (AAPC: 0.21; 95% CI: −1.19 to 1.62, *p* = 0.773. Mortality rates declined significantly from 1999 to 2014 (APC: −2.41; 95% CI: −4.20 to −0.59, *p* = 0.012), followed by a significant increase between 2014 and 2025 (APC: 3.89; 95% CI: 1.39–6.46, *p* = 0.004). In the White population, AAMRs declined from 1.98 (95% CI: 1.91–2.05) in 1999 to 1.54 (95% CI: 1.48–1.59) in 2025 (AAPC: −1.09; 95% CI: −1.93 to −0.24, *p* = 0.012). Mortality rates were stable from 1999 to 2001, followed by a significant decline between 2001 and 2009 (APC: −4.57; 95% CI: −5.42 to −3.72, *p* < 0.001) and a continued decrease through 2016 (APC: −2.07; 95% CI: −3.34 to −0.80, *p* = 0.004). Mortality rates then increased significantly from 2016 to 2020 (APC: 6.18; 95% CI: 2.25–10.27, *p* = 0.004), before declining non-significantly between 2020 and 2025. As illustrated in Table 1, **Supplemental Table 4,** Fig. 4.

**Fig. 4:**
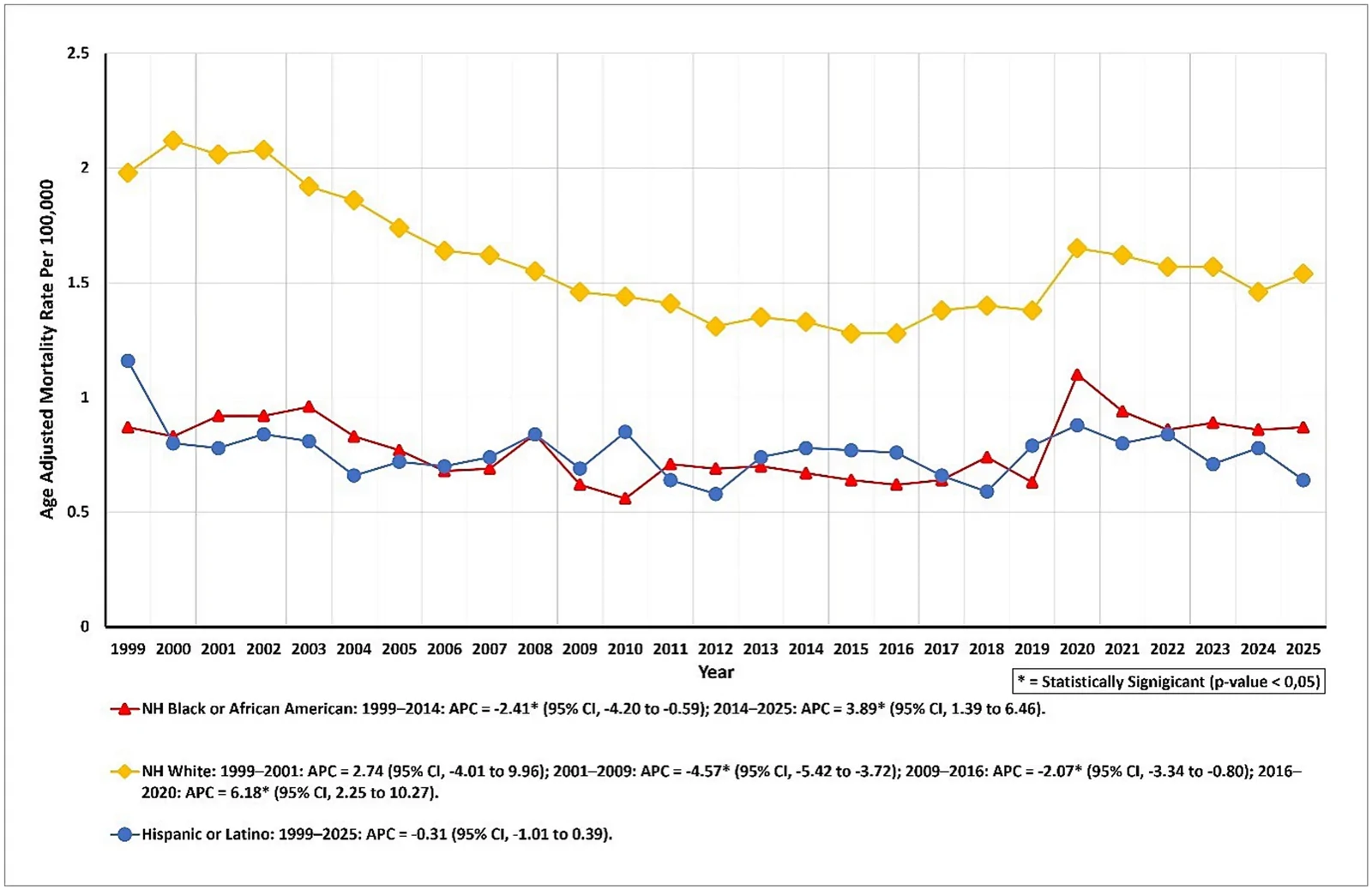
Race stratified Age-adjusted mortality rates (per 100,000) for coronary artery disease and depression or depressive-anxiety related mortality among U.S. adults aged ≥25, 1999 to 2025. APC = Annual Percentage Change, CI = Confidence Interval. *Indicates that the Annual Percentage Change (APC) is significantly different from zero at α = 0.05.

### 3.6 Urbanization

Nonmetropolitan areas had a higher mean annual AAMR (1.94) compared with metropolitan areas, which had mean annual AAMR of 1.33. In metropolitan areas, AAMRs declined from 1.76 (95% CI: 1.69–1.83) in 1999 to 1.37 (95% CI: 1.32–1.42) in 2020 (AAPC: −1.74; 95% CI: −2.75 to −0.71, *p* = 0.001). Mortality rates decreased significantly from 1999 to 2011 (APC: −4.49; 95% CI: −5.22 to −3.75, *p* < 0.001), followed by a stable trend between 2011 and 2018. From 2018 to 2020, mortality rates increased significantly (APC: 12.16; 95% CI: 2.34–22.92, *p* = 0.018). In non-metropolitan areas, AAMRs declined from 2.08 (95% CI: 1.92–2.23) in 1999 to 1.98 (95% CI: 1.84–2.11) in 2020 (AAPC: −1.16; 95% CI: −2.15 to −0.17, *p* = 0.022). Mortality rates showed a significant decline between 1999 and 2016 (APC: −2.26; 95% CI: −2.83 to −1.67, *p* < 0.001), followed by a non-significant increase from 2016 to 2020. As illustrated in Table 1 **Supplemental Table 5,** Fig. 5.

**Fig. 5:**
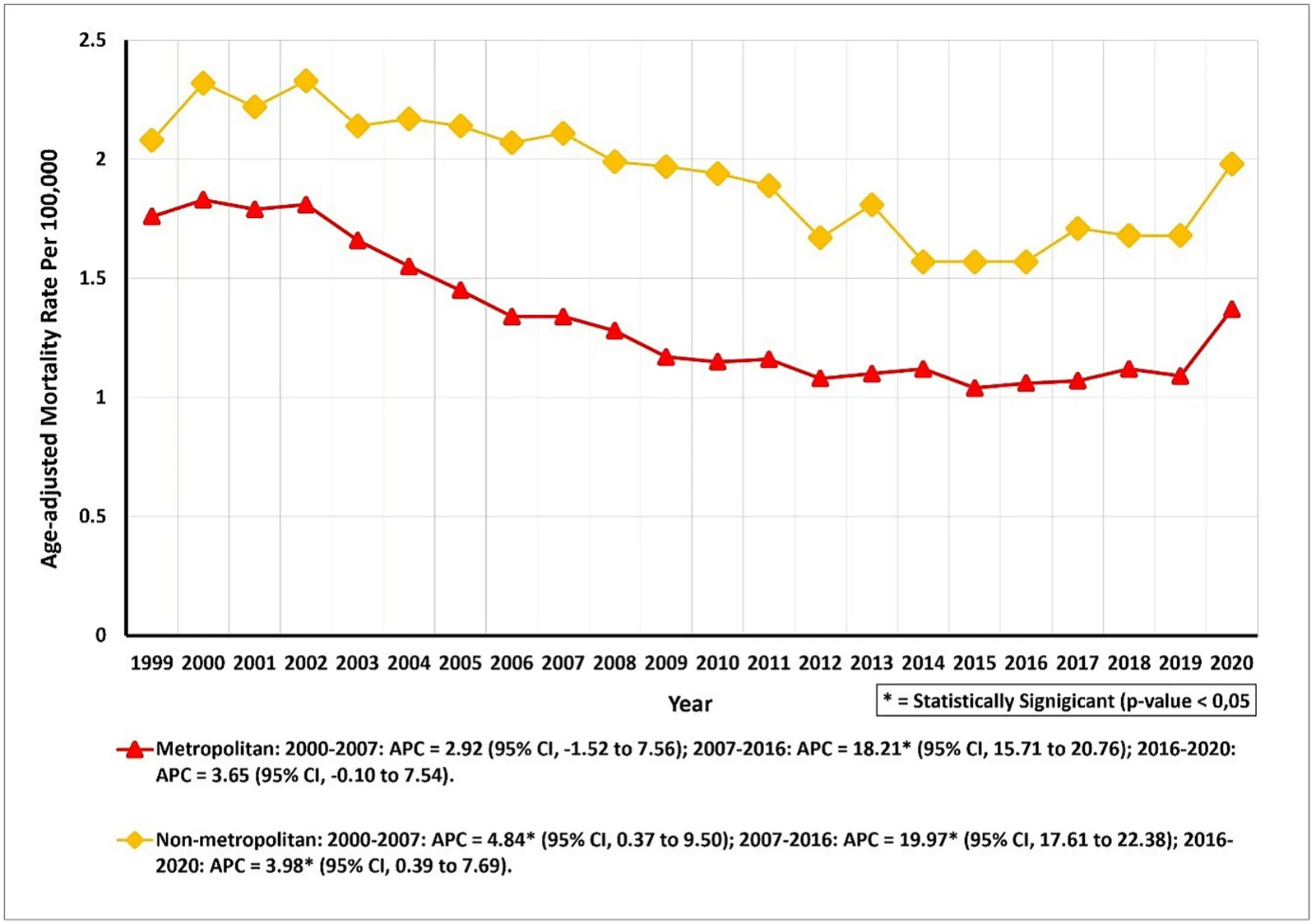
Urbanization stratified age-adjusted mortality rates per 100,000 for coronary artery disease and depression or depressive-anxiety related mortality in United States adults aged ≥25 years, 1999–2020. APC = Annual Percentage Change, CI = Confidence Interval. *Indicates that the Annual Percentage Change (APC) is significantly different from zero at α = 0.05.

### 3.7 Place of death

According to place of death, most deaths occurred in nursing home/long-term care facilities (41.57%), followed by the decedent's home (29.12%). Medical facility inpatient deaths accounted for 15.70%, while medical facility outpatient or emergency department deaths contributed 5.97%. Other places of death represented 4.49%, and hospice facilities accounted for 2.43%. A small proportion of deaths occurred as dead-on arrival (0.60%), while the place of death unknown (0.11%) and medical facility status unknown (0.05%) was least frequent. As illustrated in **Supplemental Table 6, Supplementary Fig. 1**.

### 3.8 State

From 1999 to 2020, the highest mean annual AAMR was observed in North Dakota (3.39), whereas the lowest was recorded in Nevada (0.30). States in the highest-burden percentile group included North Dakota, Vermont, Ohio, Minnesota, Iowa, Rhode Island, Nebraska, and Maine, while those in the lowest-burden percentile group included Nevada, District of Columbia, Alaska, Utah, Georgia, Arizona, Hawaii, Virginia, and Louisiana. From 2021 to 2025, the highest mean annual AAMR was observed in Wyoming (3.93), whereas the lowest was recorded in Hawaii (0.24). States in the highest-burden percentile group included Wyoming, Nebraska, Minnesota, Oregon, South Dakota, Colorado, Mississippi, Iowa, West Virginia, Indiana, Kansas, Idaho, Maryland, Ohio, and Vermont, whereas those in the lowest-burden percentile group included Hawaii, Nevada, Alabama, Utah, Massachusetts, Illinois, Connecticut, Georgia, California, and Rhode Island. These state-level patterns are presented in **Supplementary Table 7 and Supplementary Figs. 2 and 3**.

#### 3.8.1 Overall trend excluding 2025 provisional data

The updated 1999–2024 analysis showed a similar mortality pattern to the previous 1999–2025 analysis, with total deaths decreasing from 85,806 to 82,127 and the average overall AAMR remaining 1.42. Males continued to have a higher average AAMR than females (1.52 vs. 1.34). Overall AAMR declined from 1.81 to 1.28, with a slightly stronger AAPC (−1.42 vs. −1.38). The major trends remained consistent, including significant declines during 2001–2010 and 2010–2017, a marked increase during 2017–2020 (APC: 8.38), and a stronger post-2020 decline (APC: −2.84) in the updated analysis. Female mortality showed a stronger overall decline (AAPC: −1.89), whereas the male decline was no longer statistically significant (AAPC: −0.90, *p* = 0.064) compared with the previous significant result (−0.92, *p* = 0.043).

#### 3.8.2 Sensitivity analysis using F32–F33

AAMRs decreased from 1.76 (95% CI: 1.70–1.83) in 1999 to 1.26 (95% CI: 1.22–1.31) in 2024 (AAPC: −1.35; 95% CI: −2.19 to −0.51, *p* = 0.002). The AAMR showed a non-significant increase from 1999 to 2001 (APC: 3.50; 95% CI: −2.26 to 9.61, *p* = 0.215), followed by a significant decline from 2001 to 2010 (APC: −4.59; 95% CI: −5.20 to −3.98, *p* < 0.001) and a further significant decline from 2010 to 2017 (APC: −1.61; 95% CI: −2.58 to −0.62, *p* = 0.004). Mortality then increased significantly from 2017 to 2020 (APC: 8.60; 95% CI: 2.52 to 15.03, *p* = 0.009), followed by a significant decline from 2020 to 2024 (APC: −2.97; 95% CI: −4.56 to −1.36, *p* = 0.002).

## 4 Discussion

Our analysis of CDC WONDER data for 1999–2025 shows that age-adjusted mortality rates (AAMRs) for deaths in which both coronary artery disease (CAD) and depression were recorded on the death certificate declined overall. The decline was not uniform. Rates were stable from 1999 to 2001, fell steeply from 2001 to 2010 and more gradually to 2017, rose significantly between 2017 and 2020, and declined again from 2020 to 2025. This sequence parallels broader national trends in atherosclerotic cardiovascular mortality, which declined before 2018 and subsequently increased [16]. Before considering possible explanations for this pattern, an important interpretive caveat should be stated. CDC WONDER contains only information recorded on death certificates; it holds no data on risk-factor control, medication use, disease severity, depression treatment, healthcare access, or the circumstances of care. None of the factors discussed below was measured in this study. The explanations that follow are therefore offered as hypotheses consistent with the observed patterns and with the external literature. They are not mechanisms demonstrated by this analysis.

Two broad and non-exclusive classes of explanation should be considered. The first is change in the underlying risk of death among people with both conditions. The second is change in how these conditions are diagnosed, certified and coded. With respect to the first, the steep decline of the early 2000s coincides with the period of most rapid decline in ischemic heart disease mortality in the United States, which has been attributed to a combination of population-level risk-factor modification and the diffusion of evidence-based therapy, including lipid-lowering and antithrombotic therapy, timely reperfusion and revascularization, and secondary prevention such as cardiac rehabilitation [17], [18]. The attenuation of that decline after the mid-2010s has likewise been described for cardiovascular mortality generally and has been discussed in relation to the rising prevalence of obesity [19] and diabetes [20], diminishing incremental returns from risk-factor control [18], [21], and the persistent undertreatment of depression [22]. Whether any of these processes operated in the population represented by our data cannot be determined here.

With respect to the second class of explanation, our case definition requires that both conditions be recorded on the same death certificate, so secular change in the recognition and documentation of depression could alter measured rates independently of any change in true mortality risk. Both the diagnostic threshold for depression and the intensity of case-finding shifted over the study period. The US Preventive Services Task Force revised its guidance on depression screening in adults more than once during this interval. Its 2016 statement, which updated the 2009 recommendation, removed the earlier provision restricting screening to practices with staff-assisted depression care supports in place, and extended the recommendation to pregnant and postpartum women [23]. In 2023 the Task Force again recommended screening for adults and named older adults explicitly [24]. The American Heart Association issued a science advisory on depression screening, referral and treatment in coronary heart disease in 2008 [25], and screening for depression in ambulatory settings increased over a comparable period [26]. The publication of DSM-5 in 2013, which removed the bereavement exclusion for major depressive disorder, broadened the criteria under which a depressive episode could be diagnosed [27]. Greater clinical recognition of depression plausibly increases the probability that depression is listed as a contributing condition when a person with CAD dies, which would raise the measured rate without any change in underlying mortality risk. Conversely, under-recognition and under-recording earlier in the study period would depress rates in those years. These influences act in opposite directions and cannot be separated using death-certificate data. We raise these possibilities to indicate that the trends should not be read as a pure measure of change in mortality risk, rather than to explain them.

The increase observed between 2017 and 2020 overlaps the first year of the COVID-19 pandemic, a period in which excess cardiovascular mortality, deferral of outpatient cardiovascular care, and increases in psychological distress were all documented nationally [21], [28], [29], [30]. We note, however, that the increase in our data began in 2017, before the pandemic, so pandemic-related disruption cannot account for the whole of that segment. The reason for the decline observed after 2020 cannot be established from this analysis. It may reflect a genuine reduction in mortality involving these conditions; it may reflect changes in healthcare utilization or in the composition of the population at risk; it may reflect changes in certification or coding practice; or it may reflect incompleteness of the provisional records that supply the most recent years, which are subject to later revision [31]. We previously attributed this decline to improved healthcare access and the expansion of telemedicine. We have removed that attribution, as our data provide no evidence bearing on it. Continued surveillance using final mortality files will be needed to determine whether the decline is durable.

Mean annual AAMRs were higher among male than female decedents (1.52 versus 1.33), and rates declined in both sexes over the study period, though less steeply in men (AAPC −0.92) than in women (AAPC −1.81). In the broader literature the association between depressive symptoms and cardiovascular disease differs between men and women [32], and men have a higher incidence and earlier onset of obstructive CAD, more extensive atherosclerosis, and greater exposure to traditional risk factors such as smoking [33], [34]. These are plausible contributors to the higher male AAMRs observed here, although none of these characteristics is recorded in CDC WONDER. Depression is more prevalent among women and has been associated with poorer prognosis after myocardial infarction and revascularization, with inflammation, autonomic imbalance and adherence proposed as mediating pathways [32], [35]. The steeper decline in female AAMRs between 2001 and 2010 (APC −4.87) than in male AAMRs between 2002 and 2010 (APC −4.33) is compatible with, but does not demonstrate, greater benefit from increased awareness of cardiovascular disease in women and uptake of preventive treatment. Between 2017 and 2020, AAMRs rose significantly in men (APC 8.01; *p* = 0.028) and non-significantly in women (APC 7.74; *p* = 0.104). This interval coincided with documented disruption of cardiac rehabilitation delivery [36] and of mental health care [29], but the contribution of these disruptions to the increase we observed cannot be assessed with these data.

Age-specific mortality patterns showed substantial heterogeneity across the age groups analyzed. The highest average CMR was observed among adults aged ≥85 years (22.65), followed by those aged 75–84 years (6.51), 65–74 years (2.13), 55–64 years (0.88), 45–54 years (0.31), and 35–44 years (0.08). Mortality increased significantly among adults aged 35–44 years (AAPC 0.83; *p* = 0.021) and 55–64 years (AAPC 1.21; *p* < 0.001), whereas the overall trends were not statistically significant among those aged 45–54 years (AAPC 1.42; *p* = 0.054) or 65–74 years (AAPC −0.10; *p* = 0.841). In contrast, mortality declined among adults aged 75–84 years (AAPC −1.67; *p* = 0.018) and ≥ 85 years (AAPC −2.39; *p* < 0.001). These findings suggest that the overall decline in mortality was driven primarily by reductions among older age groups, while mortality increased among several younger and middle-aged groups [37].

Comparable analyses of cardiovascular mortality among adults with depression have reported declining overall mortality alongside slower improvement or unfavorable trends in some middle-aged groups [38], [39]. Earlier onset of cardiometabolic risk, psychosocial stress, and suboptimal adherence to long-term therapy have been proposed as potential contributors to less favorable mortality patterns among younger and middle-aged adults; however, these factors were not measured in our data and therefore cannot be evaluated directly. Among older adults, cumulative benefits of secondary prevention, revascularization, and cardiac rehabilitation, together with more frequent healthcare contact and greater opportunity for depressive disorders to be recognized and recorded, are plausible contributors to the observed declines [17], [37], although these mechanisms cannot be tested using death-certificate data. The increase observed among adults aged ≥85 years from 2015 to 2020 (APC 4.99; *p* = 0.021), followed by a nonsignificant decline from 2020 to 2025 (APC −2.59; *p* = 0.063), occurred during a period that included the early COVID-19 pandemic and is temporally consistent with reports of substantial excess mortality among older and frail populations during this period [30], [40]. However, COVID-19 exposure, frailty, and comorbidity burden were not ascertainable from the available mortality data.

There were marked regional differences in recorded mortality. The Midwest had the highest average AAMR (1.91), followed by the Northeast (1.36), the West (1.26) and the South (1.22). A previous national analysis of depression-related cardiovascular mortality similarly reported higher AAMRs in the Midwest and lower rates in the South and West, with a regional ordering that differs from that of cardiovascular mortality in the general population [41].

We emphasize that these are observed differences in recorded mortality. This study did not measure healthcare access, cardiometabolic risk burden, availability of mental health services, structural inequities or socioeconomic conditions, and no causal inference about regional determinants is possible from these data. Prior work has documented geographic variation in cardiovascular health, in the supply of behavioral health providers, and in cardiovascular mortality across the United States [42], [43], [44], and regional variation in health care utilization among adults with inadequate cardiovascular health has also been described [45]. Whether and to what extent such factors contribute to the differences we observed cannot be quantified here. The significant increase in the South between 2017 and 2020 (APC 14.41) and the stable trend in the West after 2015 are likewise reported as observed patterns. Possible contributors such as differences in the prevalence of obesity, diabetes and hypertension, in smoking, or in the organization and integration of health systems [42], [43], [45], [46] remain untested hypotheses in the present analysis.

Among the race and ethnicity groups for which data were reportable, average AAMRs were highest among White decedents (1.59), followed by Black or African American decedents (0.78) and Hispanic or Latino decedents (0.76), with heterogeneous trends over time. Deaths among American Indian or Alaska Native and Asian or Pacific Islander decedents were too few to be reported reliably and were not analyzed. Approximately 1284 of the 85,806 deaths (1.5%) fall outside the three groups presented, so these results do not describe the full racial and ethnic distribution of this mortality. National analyses of depression-related cardiovascular mortality report a similar ordering, with the highest rates among non-Hispanic White adults [38], [39]. Among White decedents, early declines were followed by a significant increase between 2016 and 2020 (APC 6.18) and a non-significant decline thereafter, a sequence consistent with reports of decelerating progress in ischemic heart disease mortality [18] and of rising cardiovascular mortality among adults with mental and behavioral disorders [39]. Among Black decedents, a significant decline from 1999 to 2014 (APC −2.41) was followed by a significant increase from 2014 to 2025 (APC 3.89), with no net change across the period as a whole. The literature offers several explanations for such divergence, including incomplete translation of gains in risk-factor control and acute cardiovascular care [47], differential access to high-quality cardiac and mental health services [48], and chronic psychosocial stress exposure [49]. Each is plausible, but none of these variables was measured in our study, and the present analysis cannot adjudicate between them. The non-significant decline among Hispanic or Latino decedents warrants particular caution in interpretation, since lower recorded mortality in this group has been variously attributed to a genuine survival advantage in some subgroups [50], to underdiagnosis of depression [51], and to misclassification of Hispanic ethnicity on death certificates [52]. These possibilities cannot be distinguished with these data.

Because the urbanization variable was available only for the 1999–2020 period, urban–rural comparisons cover a shorter interval than the other analyses and are not directly comparable with them. Non-metropolitan AAMRs were substantially higher on average than metropolitan AAMRs (1.94 versus 1.33), and both declined over the period, although the decline was smaller in non-metropolitan areas (AAPC −1.16) than in metropolitan areas (AAPC −1.74). Metropolitan rates increased significantly between 2018 and 2020 (APC 12.16), coinciding with the broader national reversal in cardiovascular mortality trends during this period [21]. Higher cardiovascular mortality among adults with depression in non-metropolitan areas has been reported previously [38], [41] and has been discussed in relation to the supply of cardiology and mental health specialists, travel distance and socioeconomic disadvantage [42], [43], and to the availability of cardiac rehabilitation and structured depression treatment outside metropolitan areas [53]. None of these characteristics was measured in our analysis, so we can report that the excess non-metropolitan burden persists in deaths involving both CAD and depression, but we cannot say why. The metropolitan increase in 2018–2020 is temporally consistent with reports of increased cardiovascular deaths and deferred care in urban areas during pandemic surges [28], [30] and with documented increases in psychological distress and depressive symptoms [29], but temporal coincidence alone does not establish a causal link. These patterns are consistent with continued interest in models of care that integrate cardiovascular and mental health management, and in telehealth where in-person resources are limited [54]. The effectiveness of such approaches in rural populations with co-occurring depression and CAD remains to be established prospectively.

Among deaths with both conditions recorded, the most frequently reported place of death was a nursing home or long-term care facility (41.57%), followed by the decedent's home (29.12%) and inpatient medical facilities (15.70%), with a smaller proportion occurring in hospice facilities (2.43%). Place of death is an administrative field indicating only where death occurred. It carries no information on the clinical course preceding death, functional status, care preferences, referral decisions or circumstances of care, and the interpretive weight it can bear is correspondingly limited. Studies of cardiovascular decedents more generally report that hospitals, home and long-term care facilities are common places of death, and the evidence on end-of-life care among people with serious mental illness is heterogeneous [55], [56].

The prominence of long-term care in our data may partly reflect the age structure of this group, since 72,437 of 85,805 deaths (84.4%) occurred at age 65 years or older, rather than any effect specific to co-occurring depression. We cannot determine from these data whether people with CAD and depression are more likely than others to reach advanced frailty or functional dependence before death, and that question requires patient-level study. The low proportion of deaths recorded in hospice facilities requires an additional caveat. The hospice facility category exists in CDC WONDER only for deaths occurring in 2003 and later, so hospice could not be recorded as a place of death in 1999–2002 [31]. A percentage calculated across the whole study period therefore understates the hospice proportion for the years in which the category was actually available. Subject to that caveat, the finding is consistent with literature describing later or less frequent palliative referral in cardiovascular disease than in cancer, attributed in part to prognostic uncertainty and competing clinical priorities [57], but our data contain no information on referral or prognosis and cannot confirm this, and whether co-occurring mental health conditions compound such barriers is unknown. The substantial proportion of deaths occurring at home likewise cannot be attributed to social isolation, limited caregiver support, medication non-adherence or unmonitored cardiac decompensation. Each of these has been associated with adverse cardiovascular outcomes in other study designs [35], [49], but none is recorded in CDC WONDER, and place of death alone cannot distinguish among them.

State-level results were obtained for two separate intervals, 1999–2020 and 2021–2025, drawn from different CDC WONDER files. They are therefore presented as period-specific descriptions rather than as a single continuous state-level series, and we have not combined or weighted AAMRs across the two intervals, as doing so would not be methodologically appropriate. In 1999–2020 the highest annual mean AAMR was recorded in North Dakota (3.39) and the lowest in Nevada (0.30); in 2021–2025 the highest was in Wyoming (3.93) and the lowest in Hawaii (0.24). Five states (Ohio, Vermont, Minnesota, Iowa and Nebraska) appeared in the highest-burden group in both intervals, and four (Nevada, Utah, Georgia and Hawaii) appeared in the lowest-burden group in both, while other states changed groups between intervals. Rhode Island, for example, was in the highest-burden group in 1999–2020 and the lowest-burden group in 2021–2025. Geographic clustering of depression-related cardiovascular mortality and of ischemic heart disease mortality more broadly has been described previously [38], [39], [44]. Differences in cardiometabolic risk profiles, socioeconomic conditions, healthcare infrastructure and the availability of mental health services have been proposed as explanations for such clustering [42], [43], but none of these variables was available in the present analysis, and our state-level findings should be read as descriptive rather than explanatory. Small numbers in less populous states also make state-level estimates less stable and more vulnerable to suppression, further limiting interpretation.

The clinical and public health implications of these findings should be read as proportional to a descriptive analysis of death-certificate data. Our results indicate that mortality involving both CAD and depression declined overall between 1999 and 2025 but remained unevenly distributed, and that several groups did not share equally in the improvement. These include middle-aged adults, in whom rates rose; men, in whom the decline was smaller than in women; non-metropolitan residents; Black decedents, among whom rates increased after 2014; and states that were consistently in the highest-burden group. These observations support continued national surveillance of mortality involving co-occurring cardiovascular and mental health conditions, and continued clinical attention to depression in people with CAD, which is already the subject of existing recommendations [23], [24], [25]. They do not, however, demonstrate that depression screening or treatment would reduce the mortality rates observed in this database. Our analysis contains no information on screening, diagnosis, treatment, adherence or healthcare access, and questions about the effectiveness of systematic depression detection, collaborative care and integrated cardiovascular and mental health services require patient-level and interventional study designs [17], [35], [54]. The heterogeneity we describe is best regarded as a signal for where such investigation is most needed.

### 4.1 Study limitations

This study has several important limitations, which together constrain the inferences that can be drawn.

### 4.2 Design

The analysis is descriptive and ecological. It cannot assess individual trajectories, disease severity, treatment patterns, adherence or psychosocial context, and it cannot establish causal relationships. All mechanistic explanations offered in the Discussion are hypotheses generated by reference to external literature, not findings of this study.

#### 4.2.1 Ascertainment from death certificates

CAD and depression were identified from ICD-10 codes recorded anywhere on the death certificate. Depression in particular is under-recognized clinically and under-reported on death certificates, and recording may vary by age, race and ethnicity, urbanization and certifier practice. Our counts and rates therefore almost certainly underestimate the true frequency of deaths in which both conditions were present, and any differential change in recording over time or between subgroups would bias the observed trends.

#### 4.2.2 Underlying versus contributing cause

Because both conditions were counted when listed in any multiple cause-of-death field, we cannot determine whether CAD, depression, or an unrelated condition was the underlying cause of death, nor the extent to which either condition contributed to the death.

#### 4.2.3 Temporal relationship between conditions

Death-certificate data give no information on the sequence, duration or severity of CAD and depression. We cannot determine whether depression preceded or followed the diagnosis of CAD, and no statement about directionality should be inferred from these results.

#### 4.2.4 Case definition

Depression was defined using ICD-10 codes F32, F33 and F41.2. The inclusion of F41.2 (mixed anxiety and depressive disorder) creates a broader case definition than F32 and F33 alone and captures a wider clinical spectrum, including presentations of lesser severity. In a sensitivity analysis using the narrower code set, AAMRs declined from 1.76 (95% CI 1.70–1.83) in 1999 to 1.26 (95% CI 1.22–1.31) in 2024 (AAPC −1.35; 95% CI −2.19 to −0.51; *p* = 0.002), with the same sequence of joinpoint segments as the main analysis. Absolute rates were slightly lower, as expected with a narrower definition, but the temporal pattern was unchanged.

#### 4.2.5 Provisional data for recent years

Data for 2025 are provisional. Provisional mortality records are incomplete at the time of extraction, may not include all deaths occurring in the period, and are subject to revision as additional records are received, processed and coded [31]. Estimates for the most recent year should therefore be regarded as preliminary. In a sensitivity analysis restricted to 1999–2024, the overall pattern was preserved (total deaths 82,127; average AAMR 1.42; AAPC −1.42), although the decline among men was no longer statistically significant (AAPC −0.90; *p* = 0.064) compared with the main analysis (AAPC −0.92; *p* = 0.043). Some findings are therefore sensitive to the inclusion of the provisional year.

#### 4.2.6 Differences between final and provisional files

The analysis draws on more than one CDC WONDER mortality file. The final Multiple Cause of Death file for 1999–2020 classifies race using bridged-race categories, whereas the 2018-onward final and provisional files use single-race categories. Population denominators and completeness also differ between files. Comparisons that cross the boundary between files, including race-specific comparisons, should therefore be made with caution, and apparent discontinuities may be artifacts of file transition rather than real changes in mortality.

#### 4.2.7 Different observation periods across analyses

Overall, sex-, age-, race- and region-stratified analyses cover 1999–2025; urbanization analyses cover 1999–2020, because that variable was available for that period; and state-level analyses were conducted separately for 1999–2020 and 2021–2025. Results across these strata are therefore not directly comparable, and differences between them may reflect differing observation windows rather than differing mortality experience.

#### 4.2.8 State-level estimates

For the reasons above, we have not calculated a combined or weighted state-level AAMR spanning the full study period. Any such combined estimate would average across different data files, race classifications and denominators and would not be interpretable. State-level estimates are also less stable in less populous states.

#### 4.2.9 Omitted and suppressed groups

Counts representing fewer than ten deaths are suppressed in CDC WONDER. Consequently, American Indian or Alaska Native and Asian or Pacific Islander decedents could not be analyzed, and some subgroup-by-year cells were unavailable. Our race- and ethnicity-specific findings apply only to the three groups reported and should not be generalized to the omitted populations, whose mortality experience may differ.

#### 4.2.10 Place-of-death categories are not constant across the study period

The “hospice facility” category is available in CDC WONDER only for deaths occurring in 2003 and later, and the “medical facility, status unknown” category only for 1999–2002 [31]. Percentages calculated over the full period are therefore computed across years in which some categories could not be recorded, and the hospice proportion in particular understates the proportion for the years in which hospice was an available category. Place-of-death proportions should be interpreted accordingly.

#### 4.2.11 Multiplicity and exploratory analyses

Numerous subgroup and Joinpoint analyses were conducted without adjustment for multiple comparisons. These analyses are exploratory and hypothesis-generating, and individual segment-level findings, particularly those based on short segments around inflection points such as the 2017–2020 interval, may reflect year-to-year variation, small numbers or coding change rather than durable shifts. They require confirmation in independent data.

#### 4.2.12 Unmeasured determinants

CDC WONDER contains no data on socioeconomic status, education, insurance, social support, or access to cardiology and mental health care, all of which may influence both mortality and the likelihood that either condition is recorded.

## 5 Conclusion

Age-adjusted mortality involving both coronary artery disease and depressive or depressive-anxiety disorders declined overall between 1999 and 2025, although trends were not uniform across subgroups. Rates declined substantially in the early 2000s, increased significantly from 2017 to 2020, and declined again thereafter. Mortality trends differed by age, sex, urbanization, race and ethnicity, census region, and state, with increases observed among several middle-aged age groups and among Black decedents from 2014 to 2025. Being derived from death certificates, these findings describe population-level patterns rather than causal relationships. They support continued surveillance of mortality involving co-occurring cardiovascular and mental health conditions and warrant patient-level investigation of the heterogeneity observed.

## CRediT authorship contribution statement

**Hassaan Abid:** Writing – review & editing, Writing – original draft, Methodology, Formal analysis, Conceptualization. **Sohana Memon:** Writing – review & editing, Writing – original draft. **Vishan Das:** Writing – review & editing, Writing – original draft. **Tauseef Hamid:** Writing – review & editing, Writing – original draft. **Kaneez Fatima:** Writing – review & editing, Writing – original draft. **Muhammad Mukhlis:** Writing – review & editing, Writing – original draft. **Muhammad Vazaym:** Writing – review & editing, Writing – original draft. **Rithik Kumar Khiani:** Writing – review & editing, Writing – original draft.

## Consent for publication

Not applicable.

## Ethics statement

Ethical review and approval were not required for this study because it utilized publicly available, de-identified data from the CDC WONDER database. Informed consent was not required in accordance with national legislation and institutional requirements.

## Ethics approval and consent to participate

Ethical review and approval were not required for this study because it utilized publicly available, de-identified data from the Centers for Disease Control and Prevention Wide-Ranging Online Data for Epidemiologic Research (CDC WONDER) database. Informed consent was not required in accordance with applicable national legislation and institutional requirements.

## Funding

The authors declare that no financial support was received for the research, authorship, and/or publication of this article.

## Declaration of competing interest

The authors declare that the research was conducted in the absence of any commercial or financial relationships that could be construed as a potential conflict of interest.

## Data Availability

Publicly available datasets were analyzed in this study. These data can be found in the Centers for Disease Control and Prevention Wide-ranging Online Data for Epidemiologic Research (CDC WONDER) Multiple Cause of Death database: https://wonder.cdc.gov/.
