# Supplementary material for: National trends and disparities in coronary artery disease and depression-related mortality in the United States, 1999–2025: A population-based CDC WONDER analysis
Source: Am Heart J Plus. 2026 Oct 7;71:100936. doi: 10.1016/j.ahjo.2026.100936 (PMC13677187; doi:10.1016/j.ahjo.2026.100936)
Supplement: Supplementary file 1 [file mmc1.docx]

**Supplementary Figure 1. Percentage distribution of deaths involving coronary artery disease and depressive or depressive-anxiety disorders by place of death among adults aged ≥25 years in the United States, 1999–2025.**

**Supplementary Figure 2: State stratified age-adjusted mortality rates per 100,000 for Coronary Artery Disease and Depression related mortality in United States adults aged ≥25 years, 1999-2020.**

**Supplementary Figure 3: State stratified age-adjusted mortality rates per 100,000 for Coronary Artery Disease and Depression related mortality in United States adults aged ≥25 years, 2021-2025.**

**Supplemental Table 1**: Trends in Age-Adjusted Mortality Rates and Deaths from Coronary Artery Disease and Depression among United States Adults Aged 25 and Older stratified by Overall and Sex, 1999–2025.

| **Year** | **Overall** | | **Women** | | **Men** | |
| --- | --- | --- | --- | --- | --- | --- |
|  | **Deaths** | **AAMR (95% CI)** | **Deaths** | **AAMR (95% CI)** | **Deaths** | **AAMR (95% CI)** |
| **1999** | 3215 | 1.81 (1.75–1.87) | 2007 | 1.78 (1.71–1.86) | 1208 | 1.86 (1.75–1.97) |
| **2000** | 3427 | 1.92 (1.85–1.98) | 2194 | 1.92 (1.84–2) | 1233 | 1.89 (1.78–1.99) |
| **2001** | 3375 | 1.87 (1.81–1.93) | 2150 | 1.87 (1.79–1.95) | 1225 | 1.81 (1.71–1.92) |
| **2002** | 3477 | 1.89 (1.83–1.96) | 2132 | 1.82 (1.74–1.9) | 1345 | 1.97 (1.86–2.08) |
| **2003** | 3270 | 1.75 (1.69–1.81) | 1961 | 1.66 (1.58–1.73) | 1309 | 1.83 (1.73–1.93) |
| **2004** | 3154 | 1.67 (1.61–1.73) | 1891 | 1.63 (1.55–1.7) | 1263 | 1.73 (1.64–1.83) |
| **2005** | 3048 | 1.57 (1.51–1.63) | 1785 | 1.48 (1.41–1.55) | 1263 | 1.68 (1.59–1.78) |
| **2006** | 2922 | 1.47 (1.42–1.52) | 1704 | 1.42 (1.35–1.48) | 1218 | 1.57 (1.48–1.65) |
| **2007** | 2983 | 1.48 (1.43–1.53) | 1738 | 1.39 (1.33–1.46) | 1245 | 1.55 (1.46–1.64) |
| **2008** | 2927 | 1.4 (1.35–1.45) | 1710 | 1.36 (1.29–1.42) | 1217 | 1.49 (1.41–1.58) |
| **2009** | 2792 | 1.31 (1.27–1.36) | 1622 | 1.28 (1.21–1.34) | 1170 | 1.37 (1.29–1.44) |
| **2010** | 2753 | 1.27 (1.22–1.32) | 1544 | 1.19 (1.13–1.25) | 1209 | 1.37 (1.29–1.44) |
| **2011** | 2824 | 1.26 (1.21–1.31) | 1577 | 1.17 (1.11–1.23) | 1247 | 1.36 (1.29–1.44) |
| **2012** | 2683 | 1.19 (1.14–1.23) | 1516 | 1.14 (1.08–1.2) | 1167 | 1.25 (1.18–1.32) |
| **2013** | 2820 | 1.22 (1.17–1.26) | 1548 | 1.14 (1.08–1.2) | 1272 | 1.33 (1.26–1.41) |
| **2014** | 2830 | 1.18 (1.14–1.23) | 1577 | 1.13 (1.07–1.19) | 1253 | 1.27 (1.2–1.34) |
| **2015** | 2789 | 1.15 (1.1–1.19) | 1501 | 1.05 (1–1.11) | 1288 | 1.28 (1.21–1.35) |
| **2016** | 2851 | 1.14 (1.09–1.18) | 1508 | 1.05 (1–1.11) | 1343 | 1.27 (1.2–1.34) |
| **2017** | 3030 | 1.19 (1.15–1.24) | 1588 | 1.06 (1.01–1.12) | 1442 | 1.32 (1.25–1.39) |
| **2018** | 3184 | 1.22 (1.18–1.27) | 1700 | 1.12 (1.06–1.17) | 1484 | 1.34 (1.27–1.41) |
| **2019** | 3216 | 1.22 (1.18–1.26) | 1656 | 1.08 (1.02–1.13) | 1560 | 1.38 (1.31–1.45) |
| **2020** | 3979 | 1.48 (1.43–1.52) | 2089 | 1.34 (1.28–1.4) | 1890 | 1.62 (1.54–1.69) |
| **2021** | 3687 | 1.43 (1.38–1.48) | 1913 | 1.29 (1.23–1.35) | 1774 | 1.57 (1.49–1.64) |
| **2022** | 3701 | 1.35 (1.31–1.4) | 1885 | 1.2 (1.15–1.26) | 1816 | 1.56 (1.48–1.63) |
| **2023** | 3625 | 1.32 (1.28–1.37) | 1824 | 1.19 (1.14–1.25) | 1801 | 1.51 (1.44–1.58) |
| **2024** | 3565 | 1.28 (1.24–1.33) | 1763 | 1.11 (1.05–1.16) | 1802 | 1.45 (1.38–1.52) |
| **2025** | 3679 | 1.31 (1.26–1.35) | 1848 | 1.17 (1.12–1.23) | 1831 | 1.46 (1.4–1.53) |
| **AAMR: Age Adjusted Mortality Rate, CI: Confidence interval** | | | | | | |

**Supplemental Table 2:** Trends in Crude Mortality Rates and Deaths from Coronary Artery Disease and Depression among United States Adults Aged 25 and Older stratified by Age, 1999–2025.

| **Year** | **35-44 Years** | | **45-54 Years** | | **55-64 Years** | | **65-74 Years** | | **75-84 Years** | | **85+ Years** | |
| --- | --- | --- | --- | --- | --- | --- | --- | --- | --- | --- | --- | --- |
|  | **Deaths** | **CMR (95% CI)** | **Deaths** | **CMR (95% CI)** | **Deaths** | **CMR (95% CI)** | **Deaths** | **CMR (95% CI)** | **Deaths** | **CMR (95% CI)** | **Deaths** | **CMR (95% CI)** |
| **1999** | 22 | 0.05 (0.03–0.07) | 89 | 0.24 (0.2–0.3) | 175 | 0.74 (0.63–0.85) | 435 | 2.36 (2.14–2.58) | 1129 | 9.24 (8.7–9.77) | 1365 | 32.86 (31.12–34.6) |
| **2000** | 31 | 0.07 (0.05–0.1) | 94 | 0.25 (0.2–0.31) | 193 | 0.8 (0.68–0.91) | 468 | 2.54 (2.31–2.78) | 1170 | 9.47 (8.92–10.01) | 1468 | 34.63 (32.85–36.4) |
| **2001** | 33 | 0.07 (0.05–0.1) | 111 | 0.28 (0.23–0.33) | 192 | 0.76 (0.66–0.87) | 449 | 2.44 (2.22–2.67) | 1124 | 8.93 (8.4–9.45) | 1464 | 33.95 (32.21–35.69) |
| **2002** | 32 | 0.07 (0.05–0.1) | 92 | 0.23 (0.19–0.28) | 201 | 0.75 (0.65–0.86) | 431 | 2.34 (2.12–2.57) | 1219 | 9.55 (9.01–10.09) | 1501 | 34.36 (32.62–36.1) |
| **2003** | 32 | 0.07 (0.05–0.1) | 130 | 0.32 (0.26–0.37) | 239 | 0.85 (0.75–0.96) | 404 | 2.18 (1.97–2.4) | 1111 | 8.61 (8.11–9.12) | 1346 | 30.14 (28.53–31.75) |
| **2004** | 33 | 0.08 (0.05–0.11) | 156 | 0.37 (0.32–0.43) | 236 | 0.81 (0.7–0.91) | 446 | 2.39 (2.17–2.61) | 1008 | 7.76 (7.28–8.24) | 1267 | 27.87 (26.34–29.41) |
| **2005** | 36 | 0.08 (0.06–0.11) | 123 | 0.29 (0.24–0.34) | 228 | 0.74 (0.65–0.84) | 398 | 2.11 (1.9–2.31) | 967 | 7.4 (6.93–7.86) | 1293 | 27.55 (26.05–29.05) |
| **2006** | 35 | 0.08 (0.06–0.11) | 151 | 0.35 (0.29–0.4) | 233 | 0.73 (0.64–0.82) | 413 | 2.15 (1.94–2.36) | 858 | 6.55 (6.11–6.99) | 1226 | 25.2 (23.79–26.61) |
| **2007** | 32 | 0.07 (0.05–0.11) | 127 | 0.29 (0.24–0.34) | 286 | 0.86 (0.76–0.96) | 378 | 1.92 (1.73–2.11) | 898 | 6.86 (6.41–7.31) | 1254 | 24.88 (23.51–26.26) |
| **2008** | 27 | 0.06 (0.04–0.09) | 139 | 0.31 (0.26–0.36) | 314 | 0.92 (0.82–1.02) | 391 | 1.91 (1.72–2.1) | 829 | 6.34 (5.91–6.77) | 1219 | 23.46 (22.14–24.78) |
| **2009** | 30 | 0.07 (0.05–0.1) | 155 | 0.35 (0.29–0.4) | 306 | 0.86 (0.77–0.96) | 384 | 1.81 (1.63–1.99) | 791 | 6.07 (5.65–6.5) | 1117 | 20.81 (19.59–22.03) |
| **2010** | 46 | 0.11 (0.08–0.15) | 135 | 0.3 (0.25–0.35) | 316 | 0.87 (0.77–0.96) | 390 | 1.8 (1.62–1.97) | 762 | 5.83 (5.42–6.25) | 1095 | 19.93 (18.75–21.11) |
| **2011** | 38 | 0.09 (0.07–0.13) | 145 | 0.32 (0.27–0.38) | 348 | 0.91 (0.82–1.01) | 419 | 1.86 (1.69–2.04) | 730 | 5.54 (5.14–5.94) | 1135 | 19.78 (18.63–20.93) |
| **2012** | 32 | 0.08 (0.05–0.11) | 161 | 0.36 (0.31–0.42) | 335 | 0.87 (0.78–0.96) | 434 | 1.81 (1.64–1.98) | 670 | 5.05 (4.67–5.43) | 1047 | 17.78 (16.71–18.86) |
| **2013** | 35 | 0.09 (0.06–0.12) | 157 | 0.36 (0.3–0.41) | 341 | 0.87 (0.78–0.96) | 416 | 1.65 (1.49–1.81) | 744 | 5.53 (5.14–5.93) | 1120 | 18.54 (17.45–19.63) |
| **2014** | 30 | 0.07 (0.05–0.11) | 142 | 0.33 (0.27–0.38) | 323 | 0.81 (0.72–0.89) | 559 | 2.12 (1.94–2.29) | 664 | 4.85 (4.48–5.22) | 1107 | 17.96 (16.91–19.02) |
| **2015** | 29 | 0.07 (0.05–0.1) | 144 | 0.33 (0.28–0.39) | 369 | 0.9 (0.81–0.99) | 512 | 1.86 (1.7–2.02) | 692 | 4.97 (4.6–5.34) | 1041 | 16.56 (15.55–17.56) |
| **2016** | 33 | 0.08 (0.06–0.11) | 141 | 0.33 (0.28–0.38) | 398 | 0.96 (0.87–1.05) | 551 | 1.92 (1.76–2.09) | 690 | 4.85 (4.49–5.21) | 1028 | 16.11 (15.13–17.1) |
| **2017** | 28 | 0.07 (0.05–0.1) | 140 | 0.33 (0.28–0.39) | 367 | 0.87 (0.78–0.96) | 581 | 1.96 (1.8–2.12) | 754 | 5.13 (4.76–5.49) | 1150 | 17.78 (16.75–18.81) |
| **2018** | 26 | 0.06 (0.04–0.09) | 122 | 0.29 (0.24–0.35) | 417 | 0.99 (0.89–1.08) | 590 | 1.93 (1.78–2.09) | 823 | 5.35 (4.98–5.71) | 1199 | 18.32 (17.28–19.36) |
| **2019** | 29 | 0.07 (0.05–0.1) | 114 | 0.28 (0.23–0.33) | 388 | 0.91 (0.82–1) | 648 | 2.06 (1.9–2.22) | 854 | 5.35 (4.99–5.71) | 1173 | 17.76 (16.74–18.78) |
| **2020** | 36 | 0.09 (0.06–0.12) | 144 | 0.36 (0.3–0.42) | 439 | 1.04 (0.94–1.13) | 872 | 2.68 (2.5–2.86) | 1057 | 6.42 (6.04–6.81) | 1424 | 21.39 (20.28–22.5) |
| **2021** | 41 | 0.1 (0.07–0.13) | 148 | 0.37 (0.31–0.43) | 468 | 1.11 (1.01–1.21) | 827 | 2.48 (2.31–2.65) | 1008 | 6.28 (5.89–6.67) | 1183 | 19.97 (18.83–21.11) |
| **2022** | 47 | 0.11 (0.08–0.15) | 123 | 0.31 (0.26–0.36) | 426 | 1.03 (0.93–1.12) | 802 | 2.4 (2.23–2.57) | 1072 | 6.18 (5.81–6.55) | 1220 | 18.99 (17.92–20.05) |
| **2023** | 36 | 0.08 (0.06–0.11) | 127 | 0.32 (0.26–0.38) | 422 | 1.02 (0.92–1.12) | 817 | 2.38 (2.22–2.55) | 1040 | 5.72 (5.37–6.07) | 1178 | 19.21 (18.11–20.3) |
| **2024** | 36 | 0.08 (0.06–0.11) | 117 | 0.29 (0.24–0.35) | 396 | 0.96 (0.87–1.06) | 756 | 2.16 (2–2.31) | 1121 | 5.87 (5.52–6.21) | 1132 | 17.78 (16.74–18.82) |
| **2025** | 40 | 0.09 (0.06–0.12) | 122 | 0.3 (0.25–0.36) | 379 | 0.92 (0.83–1.02) | 806 | 2.3 (2.14–2.46) | 1180 | 6.18 (5.82–6.53) | 1143 | 17.95 (16.91–18.99) |
| **CMR: Crude Mortality Rate, CI: Confidence Interval.** | | | | | | | | | | | | |

**Supplemental Table 3:** Trends in Age-Adjusted Mortality Rates and Deaths from Coronary Artery Disease and Depression among United States Adults Aged 25 and Older stratified by Census region, 1999–2025.

| **Year** | **Northeast** | | **Midwest** | | **South** | | **West** | |
| --- | --- | --- | --- | --- | --- | --- | --- | --- |
|  | **Deaths** | **AAMR (95% CI)** | **Deaths** | **AAMR (95% CI)** | **Deaths** | **AAMR (95% CI)** | **Deaths** | **AAMR (95% CI)** |
| **1999** | 778 | 2.04 (1.9–2.19) | 1090 | 2.53 (2.38–2.68) | 800 | 1.3 (1.21–1.39) | 547 | 1.59 (1.46–1.72) |
| **2000** | 757 | 1.96 (1.82–2.1) | 1177 | 2.75 (2.59–2.9) | 957 | 1.54 (1.44–1.64) | 536 | 1.54 (1.41–1.67) |
| **2001** | 732 | 1.91 (1.77–2.04) | 1096 | 2.51 (2.37–2.66) | 965 | 1.54 (1.44–1.64) | 582 | 1.61 (1.48–1.74) |
| **2002** | 735 | 1.86 (1.73–1.99) | 1248 | 2.82 (2.67–2.98) | 904 | 1.42 (1.33–1.52) | 590 | 1.6 (1.47–1.73) |
| **2003** | 674 | 1.68 (1.56–1.81) | 1114 | 2.5 (2.36–2.65) | 871 | 1.35 (1.26–1.44) | 611 | 1.62 (1.49–1.75) |
| **2004** | 630 | 1.58 (1.45–1.7) | 1090 | 2.41 (2.27–2.55) | 849 | 1.28 (1.19–1.37) | 585 | 1.5 (1.37–1.62) |
| **2005** | 613 | 1.51 (1.39–1.63) | 1061 | 2.3 (2.16–2.43) | 816 | 1.22 (1.13–1.3) | 558 | 1.39 (1.27–1.5) |
| **2006** | 519 | 1.23 (1.12–1.34) | 1027 | 2.19 (2.06–2.32) | 815 | 1.16 (1.08–1.24) | 561 | 1.35 (1.24–1.47) |
| **2007** | 526 | 1.25 (1.14–1.36) | 1088 | 2.29 (2.15–2.43) | 794 | 1.12 (1.04–1.19) | 575 | 1.36 (1.25–1.47) |
| **2008** | 511 | 1.18 (1.08–1.28) | 987 | 2.03 (1.91–2.16) | 807 | 1.1 (1.02–1.18) | 622 | 1.46 (1.34–1.57) |
| **2009** | 509 | 1.15 (1.05–1.25) | 960 | 1.96 (1.84–2.09) | 769 | 1.02 (0.94–1.09) | 554 | 1.23 (1.12–1.33) |
| **2010** | 507 | 1.14 (1.04–1.24) | 879 | 1.76 (1.65–1.88) | 834 | 1.08 (1.01–1.16) | 533 | 1.15 (1.05–1.25) |
| **2011** | 575 | 1.29 (1.19–1.4) | 856 | 1.68 (1.57–1.79) | 808 | 1.02 (0.95–1.09) | 585 | 1.23 (1.13–1.33) |
| **2012** | 555 | 1.2 (1.1–1.3) | 764 | 1.46 (1.35–1.56) | 772 | 0.94 (0.88–1.01) | 592 | 1.2 (1.1–1.3) |
| **2013** | 517 | 1.1 (1–1.2) | 785 | 1.5 (1.4–1.61) | 947 | 1.11 (1.04–1.18) | 571 | 1.14 (1.04–1.23) |
| **2014** | 552 | 1.15 (1.05–1.25) | 845 | 1.56 (1.45–1.66) | 846 | 0.97 (0.9–1.03) | 587 | 1.12 (1.03–1.21) |
| **2015** | 541 | 1.15 (1.05–1.25) | 777 | 1.44 (1.34–1.54) | 889 | 1 (0.93–1.07) | 582 | 1.07 (0.98–1.16) |
| **2016** | 524 | 1.07 (0.98–1.17) | 828 | 1.52 (1.41–1.63) | 896 | 0.98 (0.91–1.04) | 603 | 1.09 (1–1.17) |
| **2017** | 591 | 1.18 (1.08–1.28) | 885 | 1.59 (1.48–1.69) | 931 | 1.02 (0.95–1.08) | 623 | 1.09 (1.01–1.18) |
| **2018** | 637 | 1.27 (1.17–1.37) | 894 | 1.56 (1.46–1.67) | 1039 | 1.07 (1–1.13) | 614 | 1.04 (0.96–1.12) |
| **2019** | 571 | 1.15 (1.05–1.24) | 863 | 1.49 (1.39–1.59) | 1159 | 1.19 (1.12–1.26) | 623 | 1.02 (0.94–1.1) |
| **2020** | 789 | 1.53 (1.42–1.63) | 1009 | 1.74 (1.63–1.85) | 1488 | 1.47 (1.39–1.54) | 693 | 1.13 (1.05–1.22) |
| **2021** | 626 | 1.24 (1.14–1.34) | 950 | 1.74 (1.63–1.85) | 1440 | 1.48 (1.4–1.56) | 671 | 1.12 (1.03–1.21) |
| **2022** | 630 | 1.21 (1.12–1.32) | 916 | 1.6 (1.5–1.71) | 1450 | 1.41 (1.34–1.49) | 705 | 1.13 (1.04–1.22) |
| **2023** | 650 | 1.29 (1.19–1.4) | 857 | 1.49 (1.39–1.6) | 1410 | 1.37 (1.3–1.44) | 708 | 1.13 (1.04–1.21) |
| **2024** | 614 | 1.15 (1.06–1.25) | 905 | 1.53 (1.43–1.63) | 1400 | 1.31 (1.24–1.38) | 646 | 0.99 (0.91–1.07) |
| **2025** | 609 | 1.13 (1.04–1.23) | 917 | 1.55 (1.45–1.66) | 1489 | 1.37 (1.3–1.45) | 664 | 1.06 (0.98–1.15) |
| **AAMR: Age Adjusted Mortality Rate, CI: Confidence interval** | | | | | | | | |
|  | | | | | | | | |

**Supplemental Table 4:** Trends in Age-Adjusted Mortality Rates and Deaths from Coronary Artery Disease and Depression among United States Adults Aged 25 and Older stratified by Race, 1999–2025.

| **Year** | **NH Black or African American** | | **NH White** | | **Hispanic or Latino** | |
| --- | --- | --- | --- | --- | --- | --- |
|  | **Deaths** | **AAMR (95% CI)** | **Deaths** | **AAMR (95% CI)** | **Deaths** | **AAMR (95% CI)** |
| **1999** | 125 | 0.87 (0.72–1.03) | 2975 | 1.98 (1.91–2.05) | 88 | 1.16 (0.92–1.44) |
| **2000** | 122 | 0.83 (0.68–0.98) | 3216 | 2.12 (2.05–2.19) | 62 | 0.8 (0.61–1.03) |
| **2001** | 136 | 0.92 (0.76–1.08) | 3151 | 2.06 (1.99–2.13) | 65 | 0.78 (0.59–1) |
| **2002** | 134 | 0.92 (0.76–1.07) | 3234 | 2.08 (2.01–2.15) | 81 | 0.84 (0.66–1.06) |
| **2003** | 152 | 0.96 (0.81–1.12) | 3001 | 1.92 (1.85–1.98) | 77 | 0.81 (0.63–1.03) |
| **2004** | 131 | 0.83 (0.69–0.98) | 2933 | 1.86 (1.79–1.93) | 70 | 0.66 (0.51–0.85) |
| **2005** | 124 | 0.77 (0.63–0.91) | 2811 | 1.74 (1.67–1.8) | 78 | 0.72 (0.56–0.9) |
| **2006** | 118 | 0.68 (0.55–0.81) | 2695 | 1.64 (1.57–1.7) | 76 | 0.7 (0.55–0.89) |
| **2007** | 117 | 0.69 (0.56–0.82) | 2728 | 1.62 (1.56–1.68) | 91 | 0.74 (0.59–0.92) |
| **2008** | 150 | 0.84 (0.7–0.98) | 2625 | 1.55 (1.49–1.61) | 106 | 0.84 (0.68–1.01) |
| **2009** | 123 | 0.62 (0.51–0.74) | 2518 | 1.46 (1.4–1.52) | 94 | 0.69 (0.55–0.85) |
| **2010** | 104 | 0.56 (0.45–0.67) | 2493 | 1.44 (1.38–1.49) | 113 | 0.85 (0.69–1.01) |
| **2011** | 139 | 0.71 (0.59–0.84) | 2541 | 1.41 (1.36–1.47) | 103 | 0.64 (0.51–0.77) |
| **2012** | 139 | 0.69 (0.57–0.81) | 2410 | 1.31 (1.26–1.36) | 95 | 0.58 (0.46–0.71) |
| **2013** | 146 | 0.7 (0.58–0.82) | 2506 | 1.35 (1.3–1.41) | 124 | 0.74 (0.61–0.87) |
| **2014** | 150 | 0.67 (0.56–0.78) | 2497 | 1.33 (1.28–1.39) | 136 | 0.78 (0.65–0.91) |
| **2015** | 148 | 0.64 (0.53–0.74) | 2431 | 1.28 (1.22–1.33) | 154 | 0.77 (0.65–0.9) |
| **2016** | 150 | 0.62 (0.51–0.72) | 2481 | 1.28 (1.23–1.33) | 163 | 0.76 (0.63–0.88) |
| **2017** | 145 | 0.64 (0.53–0.75) | 2698 | 1.38 (1.33–1.43) | 147 | 0.66 (0.55–0.77) |
| **2018** | 191 | 0.74 (0.63–0.85) | 2794 | 1.4 (1.35–1.46) | 137 | 0.59 (0.49–0.7) |
| **2019** | 163 | 0.63 (0.53–0.73) | 2806 | 1.38 (1.33–1.43) | 182 | 0.79 (0.67–0.9) |
| **2020** | 284 | 1.1 (0.97–1.23) | 3383 | 1.65 (1.59–1.7) | 215 | 0.88 (0.76–1) |
| **2021** | 242 | 0.94 (0.82–1.07) | 3166 | 1.62 (1.57–1.68) | 204 | 0.8 (0.69–0.93) |
| **2022** | 232 | 0.86 (0.75–0.98) | 3181 | 1.57 (1.52–1.63) | 212 | 0.84 (0.73–0.97) |
| **2023** | 242 | 0.89 (0.78–1.01) | 3144 | 1.57 (1.52–1.63) | 190 | 0.71 (0.61–0.82) |
| **2024** | 243 | 0.86 (0.76–0.98) | 3042 | 1.46 (1.41–1.51) | 221 | 0.78 (0.67–0.89) |
| **2025** | 245 | 0.87 (0.76–0.99) | 3194 | 1.54 (1.48–1.59) | 189 | 0.64 (0.55–0.75) |
| **AAMR: Age Adjusted Mortality Rate, CI: Confidence interval, NH: Non-Hispanic** | | | | | | |

**Supplemental Table 5:** Trends in Age-Adjusted Mortality Rates and Deaths from Coronary Artery Disease and Depression among United States Adults Aged 25 and Older stratified by Urbanization, 1999–2020

**(Urbanization data limited to 1999-2020)**

| **Year** | **Metropolitan** | | **Non-metropolitan** | |
| --- | --- | --- | --- | --- |
|  | **Deaths** | **AAMR (95% CI)** | **Deaths** | **AAMR (95% CI)** |
| **1999** | 2511 | 1.76 (1.69–1.83) | 704 | 2.08 (1.92–2.23) |
| **2000** | 2632 | 1.83 (1.76–1.9) | 795 | 2.32 (2.16–2.48) |
| **2001** | 2610 | 1.79 (1.72–1.86) | 765 | 2.22 (2.06–2.38) |
| **2002** | 2667 | 1.81 (1.74–1.88) | 810 | 2.33 (2.17–2.49) |
| **2003** | 2527 | 1.66 (1.6–1.73) | 743 | 2.14 (1.98–2.29) |
| **2004** | 2397 | 1.55 (1.49–1.61) | 757 | 2.17 (2.02–2.32) |
| **2005** | 2287 | 1.45 (1.39–1.51) | 761 | 2.14 (1.99–2.29) |
| **2006** | 2175 | 1.34 (1.28–1.4) | 747 | 2.07 (1.92–2.22) |
| **2007** | 2212 | 1.34 (1.29–1.4) | 771 | 2.11 (1.96–2.27) |
| **2008** | 2181 | 1.28 (1.23–1.34) | 746 | 1.99 (1.85–2.14) |
| **2009** | 2045 | 1.17 (1.12–1.23) | 747 | 1.97 (1.83–2.11) |
| **2010** | 2018 | 1.15 (1.1–1.2) | 735 | 1.94 (1.8–2.08) |
| **2011** | 2099 | 1.16 (1.11–1.21) | 725 | 1.89 (1.75–2.03) |
| **2012** | 2033 | 1.08 (1.03–1.12) | 650 | 1.67 (1.54–1.8) |
| **2013** | 2103 | 1.1 (1.05–1.15) | 717 | 1.81 (1.68–1.95) |
| **2014** | 2189 | 1.12 (1.07–1.17) | 641 | 1.57 (1.45–1.7) |
| **2015** | 2149 | 1.04 (1–1.09) | 640 | 1.57 (1.45–1.7) |
| **2016** | 2190 | 1.06 (1.02–1.11) | 661 | 1.57 (1.45–1.69) |
| **2017** | 2298 | 1.07 (1.02–1.11) | 732 | 1.71 (1.59–1.84) |
| **2018** | 2467 | 1.12 (1.08–1.17) | 717 | 1.68 (1.55–1.8) |
| **2019** | 2485 | 1.09 (1.05–1.13) | 731 | 1.68 (1.56–1.81) |
| **2020** | 3109 | 1.37 (1.32–1.42) | 870 | 1.98 (1.84–2.11) |
| **AAMR: Age Adjusted Mortality Rate, CI: Confidence interval** | | | | |

**Supplemental Table 6:** Percentage distribution of place of death in the United States (1999–2025), with Coronary Artery Disease and Depression related mortality highlighted.

| **Place of Death** | **Deaths** | **% Total** |
| --- | --- | --- |
| **Medical Facility - Inpatient** | 13,466 | 15.70 |
| **Medical Facility - Outpatient or ER** | 5,123 | 5.97 |
| **Medical Facility - Dead on Arrival** | 510 | 0.59 |
| **Medical Facility - Status unknown** | 40 | 0.05 |
| **Decedent's home** | 24,980 | 29.11 |
| **Hospice facility** | 2,081 | 2.43 |
| **Nursing home/long term care** | 35,658 | 41.53 |
| **Other** | 3,856 | 4.49 |
| **Place of death unknown** | 91 | 0.11 |
| **Total** | 85,805 | 100 |

**Supplemental Table 7:** Trends in Age-Adjusted Mortality Rates and Deaths from Coronary Artery Disease and Depression among United States Adults Aged 25 and Older stratified by State, 1999–2025.

| **State** | **1999-2020** | | **2021-2025** | | **Total Deaths**  **1999-2025** |
| --- | --- | --- | --- | --- | --- |
|  | **Deaths** | **AAMR (95% CI)** | **Deaths** | **AAMR (95% CI)** |  |
| **Alabama** | 500 | 0.66 (0.6–0.72) | 95 | 0.46 (0.37–0.57) | 595 |
| **Alaska** | 54 | 0.78 (0.57–1.04) | suppressed | suppressed | 54 |
| **Arizona** | 607 | 0.62 (0.57–0.67) | 280 | 0.87 (0.78–0.99) | 887 |
| **Arkansas** | 539 | 1.15 (1.05–1.25) | 223 | 1.81 (1.58–2.07) | 762 |
| **California** | 7231 | 1.39 (1.35–1.42) | 1218 | 0.81 (0.76–0.85) | 8449 |
| **Colorado** | 810 | 1.25 (1.17–1.34) | 515 | 2.48 (2.27–2.71) | 1325 |
| **Connecticut** | 725 | 1.11 (1.03–1.19) | 93 | 0.54 (0.44–0.68) | 818 |
| **Delaware** | 128 | 0.86 (0.71–1.01) | 41 | 0.88 (0.63–1.22) | 169 |
| **District of Columbia** | 50 | 0.6 (0.44–0.79) | 24 | 1.14 (0.73–1.71) | 74 |
| **Florida** | 2957 | 0.78 (0.75–0.81) | 1378 | 1.16 (1.1–1.23) | 4335 |
| **Georgia** | 794 | 0.66 (0.62–0.71) | 223 | 0.57 (0.5–0.66) | 1017 |
| **Hawaii** | 189 | 0.8 (0.69–0.92) | 16 | 0.24 (0.14–0.42) | 205 |
| **Idaho** | 280 | 1.25 (1.1–1.4) | 143 | 1.94 (1.64–2.3) | 423 |
| **Illinois** | 2538 | 1.27 (1.22–1.32) | 293 | 0.54 (0.48–0.61) | 2831 |
| **Indiana** | 1649 | 1.64 (1.56–1.72) | 539 | 2.01 (1.85–2.19) | 2188 |
| **Iowa** | 1354 | 2.36 (2.24–2.49) | 320 | 2.24 (2–2.5) | 1674 |
| **Kansas** | 770 | 1.63 (1.52–1.75) | 214 | 1.84 (1.59–2.11) | 984 |
| **Kentucky** | 862 | 1.31 (1.22–1.4) | 202 | 1.12 (0.97–1.3) | 1064 |
| **Louisiana** | 551 | 0.83 (0.76–0.9) | 190 | 1.05 (0.91–1.22) | 741 |
| **Maine** | 539 | 2.19 (2–2.37) | 97 | 1.4 (1.13–1.73) | 636 |
| **Maryland** | 1409 | 1.65 (1.57–1.74) | 507 | 1.99 (1.82–2.18) | 1916 |
| **Massachusetts** | 1150 | 0.99 (0.93–1.05) | 154 | 0.5 (0.42–0.59) | 1304 |
| **Michigan** | 2252 | 1.4 (1.34–1.46) | 318 | 0.74 (0.66–0.83) | 2570 |
| **Minnesota** | 2341 | 2.75 (2.64–2.86) | 761 | 3.24 (3.01–3.49) | 3102 |
| **Mississippi** | 502 | 1.15 (1.05–1.25) | 275 | 2.39 (2.11–2.7) | 777 |
| **Missouri** | 1702 | 1.71 (1.63–1.79) | 345 | 1.31 (1.18–1.46) | 2047 |
| **Montana** | 232 | 1.31 (1.14–1.48) | 68 | 1.38 (1.06–1.78) | 300 |
| **Nebraska** | 670 | 2.15 (1.99–2.32) | 290 | 3.73 (3.31–4.2) | 960 |
| **Nevada** | 116 | 0.35 (0.29–0.42) | 57 | 0.49 (0.37–0.64) | 173 |
| **New Hampshire** | 359 | 1.66 (1.49–1.83) | 90 | 1.33 (1.07–1.66) | 449 |
| **New Jersey** | 2213 | 1.53 (1.46–1.59) | 463 | 1.15 (1.05–1.27) | 2676 |
| **New Mexico** | 315 | 1.05 (0.93–1.17) | 109 | 1.19 (0.97–1.45) | 424 |
| **New York** | 3268 | 1 (0.96–1.03) | 1349 | 1.53 (1.45–1.61) | 4617 |
| **North Carolina** | 2057 | 1.46 (1.39–1.52) | 675 | 1.56 (1.44–1.68) | 2732 |
| **North Dakota** | 427 | 3.39 (3.06–3.72) | 68 | 2.14 (1.65–2.76) | 495 |
| **Ohio** | 6115 | 3.15 (3.07–3.23) | 944 | 1.87 (1.75–2) | 7059 |
| **Oklahoma** | 935 | 1.6 (1.5–1.71) | 288 | 1.92 (1.7–2.16) | 1223 |
| **Oregon** | 1139 | 1.78 (1.67–1.88) | 478 | 2.6 (2.37–2.85) | 1617 |
| **Pennsylvania** | 4269 | 1.77 (1.72–1.83) | 784 | 1.3 (1.21–1.39) | 5053 |
| **Rhode Island** | 459 | 2.28 (2.07–2.49) | 32 | 0.64 (0.44–0.93) | 491 |
| **South Carolina** | 896 | 1.28 (1.2–1.37) | 413 | 1.86 (1.69–2.06) | 1309 |
| **South Dakota** | 319 | 2.15 (1.91–2.39) | 92 | 2.54 (2.03–3.15) | 411 |
| **Tennessee** | 1578 | 1.63 (1.55–1.71) | 466 | 1.65 (1.5–1.81) | 2044 |
| **Texas** | 4343 | 1.46 (1.41–1.5) | 1497 | 1.58 (1.5–1.66) | 5840 |
| **Utah** | 185 | 0.64 (0.55–0.73) | 52 | 0.56 (0.42–0.73) | 237 |
| **Vermont** | 361 | 3.31 (2.97–3.65) | 67 | 2.1 (1.62–2.72) | 428 |
| **Virginia** | 1135 | 1 (0.95–1.06) | 513 | 1.47 (1.35–1.61) | 1648 |
| **Washington** | 1589 | 1.61 (1.53–1.69) | 356 | 1.14 (1.02–1.27) | 1945 |
| **West Virginia** | 720 | 2.16 (2–2.32) | 179 | 2.1 (1.8–2.46) | 899 |
| **Wisconsin** | 1186 | 1.26 (1.18–1.33) | 361 | 1.41 (1.27–1.57) | 1547 |
| **Wyoming** | 180 | 2.24 (1.91–2.57) | 96 | 3.93 (3.17–4.85) | 276 |
